# Enhancing Patient Selection in Sepsis Clinical Trials Design Through an AI Enrichment Strategy: Algorithm Development and Validation

**DOI:** 10.2196/54621

**Published:** 2024-09-04

**Authors:** Meicheng Yang, Jinqiang Zhuang, Wenhan Hu, Jianqing Li, Yu Wang, Zhongheng Zhang, Chengyu Liu, Hui Chen

**Affiliations:** 1 State Key Laboratory of Digital Medical Engineering School of Instrument Science and Engineering Southeast University Nanjing China; 2 Emergency Intensive Care Unit (EICU) The Affiliated Hospital of Yangzhou University Yangzhou University Yangzhou China; 3 Key Laboratory of Big Data Analysis and Knowledge Services of Yangzhou City Yangzhou University Yangzhou China; 4 Jiangsu Provincial Key Laboratory of Critical Care Medicine, Department of Critical Care Medicine, Zhongda Hospital School of Medicine Southeast University Nanjing China; 5 School of Biomedical Engineering and Informatics Nanjing Medical University Nanjing China; 6 Department of Emergency Medicine, Key Laboratory of Precision Medicine in Diagnosis and Monitoring Research of Zhejiang Province Sir Run Run Shaw Hospital Zhejiang University School of Medicine Hangzhou China

**Keywords:** sepsis, enrichment strategy, disease progression trajectories, artificial intelligence, predictive modeling, conformal prediction

## Abstract

**Background:**

Sepsis is a heterogeneous syndrome, and enrollment of more homogeneous patients is essential to improve the efficiency of clinical trials. Artificial intelligence (AI) has facilitated the identification of homogeneous subgroups, but how to estimate the uncertainty of the model outputs when applying AI to clinical decision-making remains unknown.

**Objective:**

We aimed to design an AI-based model for purposeful patient enrollment, ensuring that a patient with sepsis recruited into a trial would still be persistently ill by the time the proposed therapy could impact patient outcome. We also expected that the model could provide interpretable factors and estimate the uncertainty of the model outputs at a customized confidence level.

**Methods:**

In this retrospective study, 9135 patients with sepsis requiring vasopressor treatment within 24 hours after sepsis onset were enrolled from Beth Israel Deaconess Medical Center. This cohort was used for model development, and 10-fold cross-validation with 50 repeats was used for internal validation. In total, 3743 patients with sepsis from the eICU Collaborative Research Database were used as the external validation cohort. All included patients with sepsis were stratified based on disease progression trajectories: rapid death, recovery, and persistent ill. A total of 148 variables were selected for predicting the 3 trajectories. Four machine learning algorithms with 3 different setups were used. We estimated the uncertainty of the model outputs using conformal prediction (CP). The Shapley Additive Explanations method was used to explain the model.

**Results:**

The multiclass gradient boosting machine was identified as the best-performing model with good discrimination and calibration performance in both validation cohorts. The mean area under the receiver operating characteristic curve with SD was 0.906 (0.018) for rapid death, 0.843 (0.008) for recovery, and 0.807 (0.010) for persistent ill in the internal validation cohort. In the external validation cohort, the mean area under the receiver operating characteristic curve (SD) was 0.878 (0.003) for rapid death, 0.764 (0.008) for recovery, and 0.696 (0.007) for persistent ill. The maximum norepinephrine equivalence, total urine output, Acute Physiology Score III, mean systolic blood pressure, and the coefficient of variation of oxygen saturation contributed the most. Compared to the model without CP, using the model with CP at a mixed confidence approach reduced overall prediction errors by 27.6% (n=62) and 30.7% (n=412) in the internal and external validation cohorts, respectively, as well as enabled the identification of more potentially persistent ill patients.

**Conclusions:**

The implementation of our model has the potential to reduce heterogeneity and enroll more homogeneous patients in sepsis clinical trials. The use of CP for estimating the uncertainty of the model outputs allows for a more comprehensive understanding of the model’s reliability and assists in making informed decisions based on the predicted outcomes.

## Introduction

Sepsis, defined as a dysregulated immune response to infection that leads to acute organ dysfunction, persistently stands as one of the leading causes of mortality worldwide [1,2]. Sepsis is a heterogeneous syndrome, underscored by various infection sites, pathogens, and a vast, multidimensional array of clinical and biological features [3], concomitant with the between-patient variability in response to treatment, which might account for the absence of benefit in most randomized controlled trials (RCTs) assessing various therapies in sepsis [4]. The targeted inclusion of more homogeneous patient populations shows great promise and is an emerging trend in clinical trials [5].

Clinical trial enrichment strategies typically encompass prognostic and predictive enrichment. PaO_2_/FiO_2_ ratio has been used to identify patients with high-risk acute respiratory distress syndrome or those more likely to respond to prone positioning [6,7]. Combined variables can enhance the accuracy of patient stratification, thereby improving the efficacy of clinical trial enrichment [8]. Several phenotypes of sepsis have been identified through various combinations of clinical features, with both prognostic and predictive enrichment [9,10]. However, these phenotypes brought another layer of complexity due to their heterogeneity and were not originally conceptualized to improve the stratification of homogeneous patient groups in sepsis clinical trials. Patients with sepsis are often accompanied by circulation dysfunction and require the use of vasopressors. Tracking how vasopressor administration changes could help identify those likely to benefit most from treatment. Some may get worse quickly and die, others might slowly improve, but some could have a longer disease progression and might gain more from clinical drug trials. Therefore, developing an artificial intelligence (AI) model to predict which patients with sepsis will have a longer course is key to making clinical trials more effective [11].

AI has now facilitated the identification of homogeneous subgroups, allowing rapid selection of target patients for potential enrollment in further RCTs [12]. However, a significant challenge in AI modeling lies in quantifying the reliability of the model predictions for new patients, particularly when data extend beyond the original training domain. In addition, most AI models provide binary point predictions, essentially yes or no, without assessing the reliability of these predictions. However, when implementing AI models in high-risk environments, it is critical to incorporate uncertainty quantification to minimize the occurrence of unanticipated model failures such as the risk of automation bias [13].

This study aimed to develop and validate a novel data-driven AI methodology for predicting patients with sepsis who are likely to have a sufficiently long disease course, enabling the enrollment of homogeneous patient populations in clinical trials. The AI model was expected to provide interpretable factors and confidence measures to monitor predictions and identify uncertain predictions at a customized confidence level for human review, so as to enroll reliable persistent ill patients.

## Methods

### Study Design and Setting

This study used data from 2 distinct databases: the Medical Information Mart for Intensive Care Database-IV (MIMIC-IV) [14] and the eICU Collaborative Research Database (eICU-CRD) [15]. MIMIC-IV contained critical care data for 73,181 patients admitted to the ICUs at Beth Israel Deaconess Medical Center between 2008 and 2019. As a large multicenter intensive care unit (ICU) database, eICU-CRD collected more than 200,000 ICU admissions from 335 units in 208 hospitals across the United States from 2014 to 2015. This study followed the TRIPOD (Transparent Reporting of a Multivariable Prediction Model for Individual Prognosis or Diagnosis) checklist (Multimedia Appendix 1).

### Ethical Considerations

The MIMIC-IV and eICU-CRD, which had been made publicly accessible, received ethical approval from the institutional review boards at Beth Israel Deaconess Medical Center and the Massachusetts Institute of Technology, in alignment with the principles outlined in the Declaration of Helsinki. The approval also encompassed a waiver of the need for informed consent, since all protected health information within the databases was deidentified [14,15]. Access to these databases was provided after completion of a training program in human research ethics and signing of a data use agreement with PhysioNet, earning a certification number of 27252652.

### Study Population

For inclusion in our study, patients were required to meet the sepsis diagnosis and initiate vasopressor treatment within 24 hours after identifying sepsis. In MIMIC-IV, sepsis was defined according to the Sepsis-3 criteria, known or suspected infection, and Sequential Organ Failure Assessment (SOFA) score ≥2 points [1]. The following 2 time points were specified to define the onset time of sepsis *t*_sepsis_ [16]: (1) *t*_suspicion_: clinical suspicion of infection as determined by the earlier timestamp of intravenous (IV) antibiotics administration and cultures acquired within a specific timeframe. If IV antibiotics were given first, the cultures must have been obtained within 24 hours. If cultures were obtained first, then antibiotics must have been subsequently ordered within 72 hours. (2) *t*_SOFA_: the occurrence of organ failure as identified by a 2-point increase in the SOFA score within a 24-hour period. *t*_sepsis_ is the earlier of *t*_suspicion_ and *t*_SOFA_, as long as *t*_SOFA_ occurs no more than 24 hours before or 12 hours after *t*_suspicion_; otherwise, the patient will not be diagnosed as sepsis. Specifically, if *t*_suspicion_–24 hours≤*t*_SOFA_≤*t*_suspicion_+12 hours, then *t*_sepsis_=min (*t*_suspicion_, *t*_SOFA_). In eICU-CRD, patients with sepsis were identified based on the admission diagnosis, with the sepsis identification defined as the time of ICU admission. To be as close as possible to the sepsis onset defined in eICU-CRD, only patients with sepsis onset within 24 hours of ICU admission were included in MIMIC-IV. Exclusion criteria included patients younger than 18 years, those who died within the first 24 hours of sepsis onset, or ICU stay of less than 24 hours. For patients with multiple ICU admissions, only the first admission was considered.

### Trajectories of Sepsis and Candidate Predictors

We hypothesized that patients with sepsis were recruited into RCTs within 24 hours of identification. Following the enrollment, all patients with sepsis were stratified based on their disease progression trajectories: “rapid death” included patients who expired within 48 hours after enrollment, “recovery” included patients who liberated from vasopressor support within 48 hours after enrollment and maintained for at least 24 hours, and “persistent ill” included patients who necessitated ongoing vasopressor support within 48 hours after enrollment.

The selection of candidate predictors incorporated both static and time-varying variables. Time-varying variables were extracted within the 24-hour period between identification and enrollment. Any outliers were identified and subsequently excluded according to the criteria defined in Multimedia Appendix 2. For time-varying variables with multiple measurements during the 24-hour duration, we included the maximum, minimum, median, and coefficient of variation (SD/mean) values for analysis. This resulted in a total of 148 features.

### Predictive Model Development and Explanation

**Figure 1:**
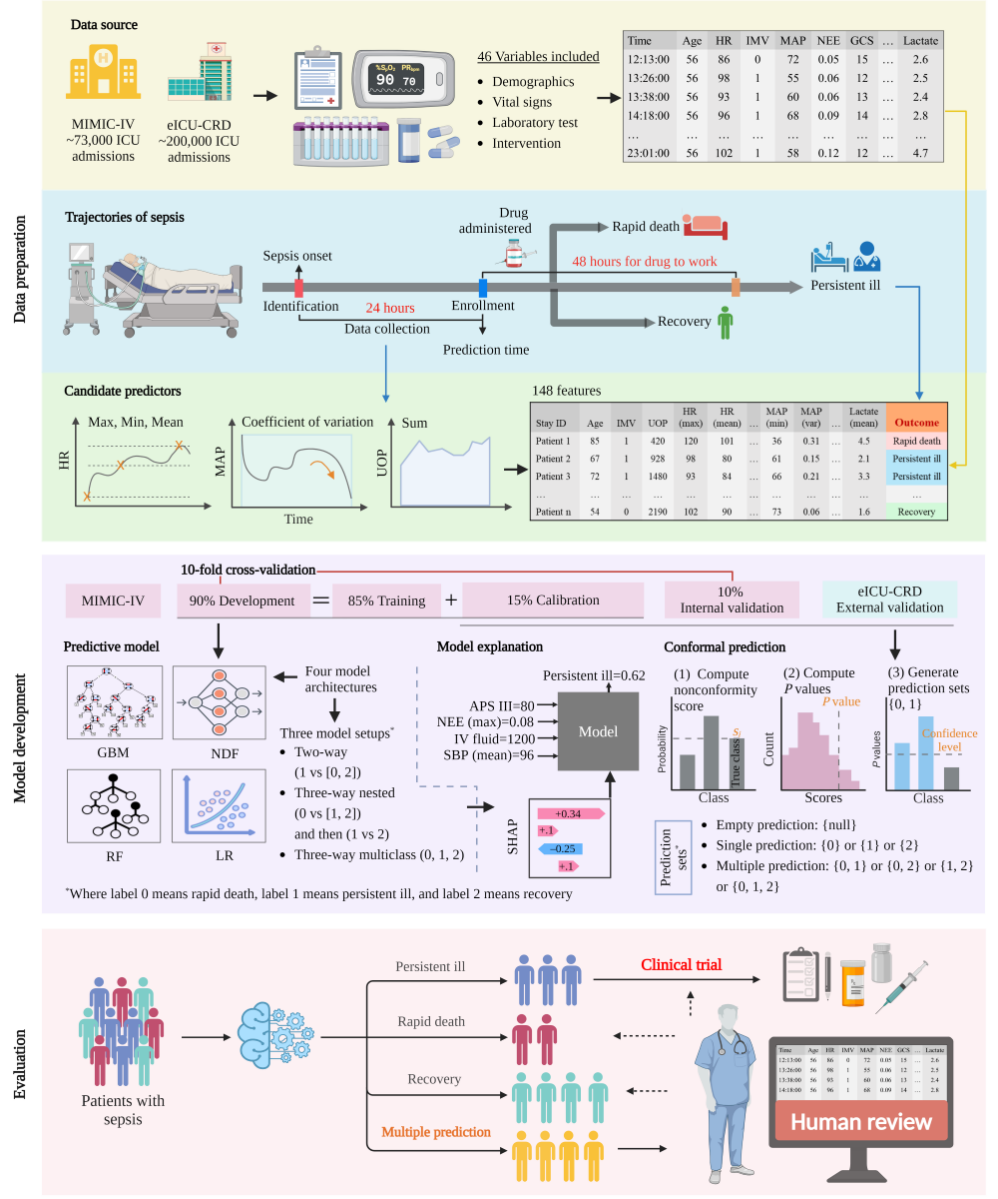
Workflow of the study. For the multiple predictions in evaluation, it means that the prediction was uncertain, and the artificial intelligence model could not distinguish between the possible class labels, thus awaiting the human review in order to result in a single class prediction. APS: Acute Physiology Score; eICU-CRD: eICU Collaborative Research Database; GBM: gradient boosting machine; GCS: Glasgow Coma Scale; HR: heart rate; ICU: intensive care unit; IMV: invasive mechanical ventilation; IV: intravenous; LR: logistic regression; MAP: mean arterial pressure; MIMIC-IV: Medical Information Mart for Intensive Care Database-IV; NDF: neural decision forest; NEE: norepinephrine equivalence; RF: random forest; SBP: systolic blood pressure; SHAP: Shapley Additive Explanations; UOP: urine output.

The MIMIC-IV data set was used for model development, and 10-fold cross-validation with 50 repeats was used for internal validation (Figure 1). The eICU-CRD was used for the retrospective external validation. A total of 12 combinations of model architectures and setups were used (Figure 1). The 4 specific architectures evaluated were gradient boosting machine (GBM) [17], neural decision forest (NDF) [18], random forest (RF), and logistic regression (LR; Multimedia Appendix 3). GBM, RF, and LR were constructed with reference to the research of Schwager et al [11] for predicting acute respiratory distress syndrome trajectories. In addition, these 3 models were commonly used in critical illness prediction tasks using electronic health record (EHR) data [19-21]. However, they were all traditional machine learning models, so we developed a deep learning model for comparison, that is the NDF, which has also been shown to be effective for disease prediction using EHR data [22].

These model architectures were used in 3 different setups, namely, the 2-way (modeling persistent ill and rapid death or recovery), 3-way nested (first predicting rapid death versus persistent ill or recovery, then classifying persistent ill or recovery), and 3-way multiclass (generating probabilities for each trajectory simultaneously) configurations. For missing data, a mean imputation method was used for NDF, RF, and LR, while GBM did not require imputation.

We used Shapley Additive Explanations [23] values to explain the best model among the 12 constructed models. In addition, we examined the performance of the lightweight model using the feature subset in 3 ways (Multimedia Appendix 3): retraining the model using only the 15 most important features (measured by Shapley Additive Explanations), using the Boruta feature selection method, or using only the maximum norepinephrine equivalence (NEE), that is, NEE (max).

### Conformal Prediction

To estimate the uncertainty of the model outputs, we used the conformal prediction (CP) framework built on top of the prediction algorithm (Multimedia Appendix 3). The CP is a method that can be mathematically guaranteed to make reliable predictions at a user-specified desired error rate for unknown samples that differ from the training data [13]. We specifically implemented a Mondrian CP to handle the prediction tasks with unbalanced data and worked on a class basis to ensure the desired error rate within each class. For the 3 trajectories of the patients with sepsis, the possible prediction sets are single predictions of {0}, {1}, {2}; multiple predictions of {0, 1}, {0, 2}, {1, 2}, {0, 1, 2}; and the empty prediction of {null}, where label 0 means rapid death, label 1 means persistent ill, and label 2 means recovery. We further split the development set into a training set to train the AI models and a calibration set (Figure 1) to develop the Mondrian CP and also to tune the model hyperparameters using a Bayesian optimizer [24].

### Statistical Analysis

Univariate analysis was performed using logistic regression and was performed by associating each variable with three trajectories: (1) rapid death versus recovery or persistent ill, (2) persistent ill versus recovery or rapid death, and (3) recovery versus rapid death or persistent ill. Coefficients and CIs were determined on the logit scale and then transformed through exponentiation to provide estimates and 95% CIs for the odds ratios.

The discrimination performance of the model was assessed using the area under the receiver operating characteristic curve (AUROC) and the area under the precision-recall curve. Calibration was performed using calibration curves and the Brier score. We mainly used the AUROC value to identify the model architecture and setup with the best performance for predicting persistent ill. Metrics such as true positive rate (TPR), positive predictive value (PPV), and the *F*-0.5 score (a composite of TPR and PPV that gives more weight to PPV) were also calculated. Cutoff values were chosen to maximize the F-0.5 score for the 2-way and 3-way nested setup. For the multiclass setup, the predicted outcome was obtained with the class that had the highest probability. These metrics were reported as mean (SD) for both the internal validation and external validation.

Since the conformal predictor could generate multiple or empty predictions, we could not calculate the TPR and PPV of the conformal predictor directly. To evaluate the effectiveness of the conformal predictor and assess its performance, we randomly select 1 model with the optimal architecture from the 10-fold cross-validation with 50 repeats as an example. We evaluated efficiency, which measures the proportion of all predictions that yield a single correct prediction, and the validity (error rate), representing the proportion of all predictions that did not surpass the predetermined significance level [13].

## Results

### Study Population

In the MIMIC-IV, 9135 patients met the inclusion and exclusion criteria. In the eICU-CRD, 3743 patients with sepsis were identified and classified as external validation cohort (Multimedia Appendix 4). The clinical characteristics of included patients are described in Table 1. In the MIMIC-IV, the median age was 68 (IQR 58-78) years, with a median SOFA score of 4 (IQR 3-5). The median NEE was 0.12 (IQR 0.07-0.25) μg/kg/minute, and the in-hospital mortality was 16.4% (n=1501). For the eICU-CRD, the median NEE was 0.15 (IQR 0.07-0.34) μg/kg/minute, and the in-hospital mortality was 22.9% (n=857). Univariate analysis was used to characterize the factors distinguishing the subpopulations of the 3 trajectories (Multimedia Appendix 5).

**Table 1:** Characterization of cohorts.

Characteristics		MIMIC-IV^a^ (n=9135)	eICU-CRD^b^ (n=3743)	*P* value	
Age (years), median (IQR)		68 (58-78)	68 (58-79)	.48	
Male, n (%)		5482 (60)	1887 (50.4)	<.001	
BMI (kg/m^2^), median (IQR)		27.7 (24.1-32.7)	27.3 (23.0-33.1)	<.001	
Emergency admission, n (%)		4635 (50.7)	2426 (64.8)	<.001	
Pulmonary infection, n (%)		3643 (39.9)	1308 (34.9)	<.001	
Charlson comorbidity index, median (IQR)		6 (4-8)	—^c^	—	
SOFA^d^ score, median (IQR)		4 (3-5)	7 (5-10)	<.001	
NEE^e^ (max) (μg/kg/min), median (IQR)		0.12 (0.07-0.25)	0.15 (0.07-0.34)	<.001	
Use of CRRT^f^, n (%)		515 (5.6)	80 (2.1)	<.001	
**Trajectories of sepsis, n (%)**					<.001
	Recovery	5835 (63.9)	2151 (57.5)		
	Persistent ill	2937 (32.1)	1283 (34.3)		
	Rapid death	363 (4)	309 (8.2)		
In-hospital mortality, n (%)		1501 (16.4)	857 (22.9)	<.001	
28-day mortality, n (%)		1386 (15.2)	—	—	

^a^MIMIC-IV: Medical Information Mart for Intensive Care Database-IV.

^b^eICU-CRD: eICU Collaborative Research Database.

^c^Not available.

^d^SOFA: Sequential Organ Failure Assessment.

^e^NEE: norepinephrine equivalence.

^f^CRRT: continuous renal replacement therapy.

### Trajectories of Sepsis

**Figure 2:**
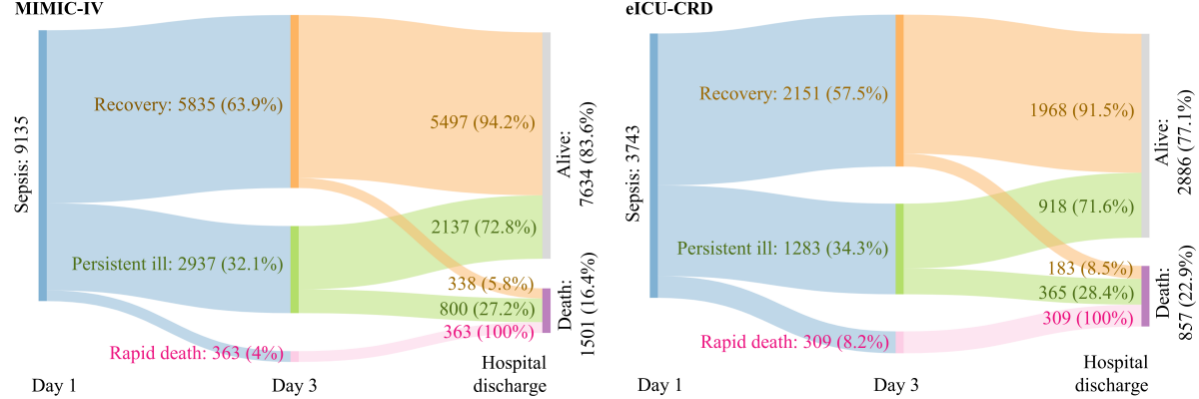
Trajectories of patients with sepsis. eICU-CRD: eICU Collaborative Research Database; MIMIC-IV: Medical Information Mart for Intensive Care Database-IV.

**Figure 3:**
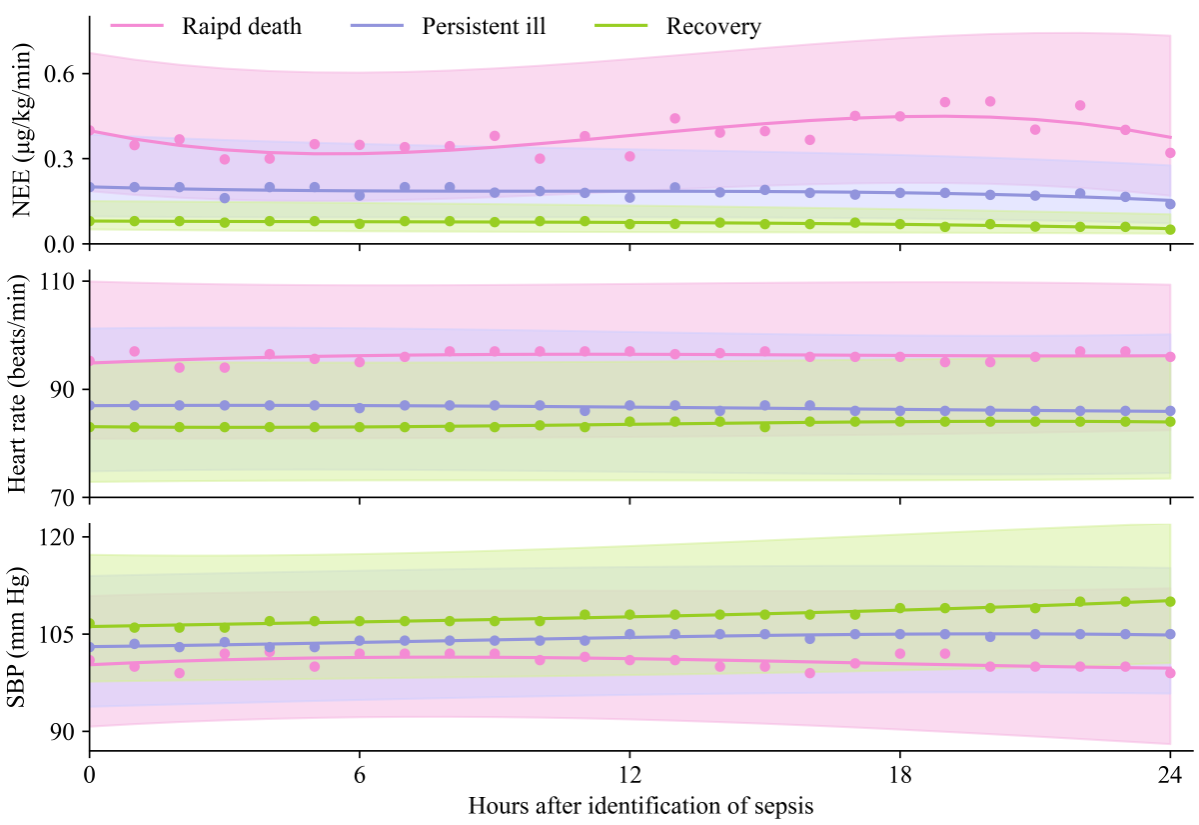
Trends of typical parameters over time according to sepsis trajectories 24 hours after identification. Points represent the median parameter values in an hourly interval. The intervals represent the 25th and 75th percentiles of the parameter values. Cubic polynomial smoothing splines highlight trends. NEE: norepinephrine equivalence; SBP: systolic blood pressure.

Patients in the MIMIC-IV exhibited the following outcomes within a 48-hour period after enrollment: 5835 (63.9%) patients experienced recovery, 2937 (32.1%) patients remained persistent ill, and 363 (4%) patients experienced rapid death. In the external validation cohort, that is, eICU-CRD, 2151 (57.5%) patients experienced recovery, 1283 (34.3%) patients remained persistent ill, and 309 (8.2%) patients experienced rapid death. In patients who recovered, the in-hospital mortality rates in the MIMIC-IV and eICU-CRD were 5.8% (n=338) and 8.5% (n=183), respectively. For patients with persistent ill, the in-hospital mortality rates in the MIMIC-IV and eICU-CRD were 27.2% (n=800) and 28.4% (n=365), respectively (Figure 2). Compared to patients with recovery or persistent ill, patients with rapid death had sustained high NEE, high heart rate, and low systolic blood pressure (SBP; Figure 3).

### Predictive Performance of AI Model

Of the 4 machine learning architectures in 3 model setups for predicting persistent ill on internal validation, GBM had the highest consistent performance of mean AUROC (SD) ranging from 0.806 (0.010) to 0.807 (0.010) compared to NDF ranging from 0.779 (0.013) to 0.802 (0.011), RF ranging from 0.791 (0.013) to 0.795 (0.010), and LR ranging from 0.719 (0.031) to 0.790 (0.007; Multimedia Appendix 6). Of the 3 model setups, the 3-way multiclass with GBM performed best with the mean AUROC of 0.807 (SD 0.010). In addition, the multiclass setup could also provide a probability risk score for each trajectory, with a mean AUROC (SD) of 0.906 (0.018) for rapid death and 0.843 (0.008) for recovery. In the external validation cohort, the mean AUROCs (SDs) were 0.878 (0.003) for rapid death, 0.764 (0.008) for recovery, and 0.696 (0.007) for persistent ill (Multimedia Appendix 7).

**Figure 4:**
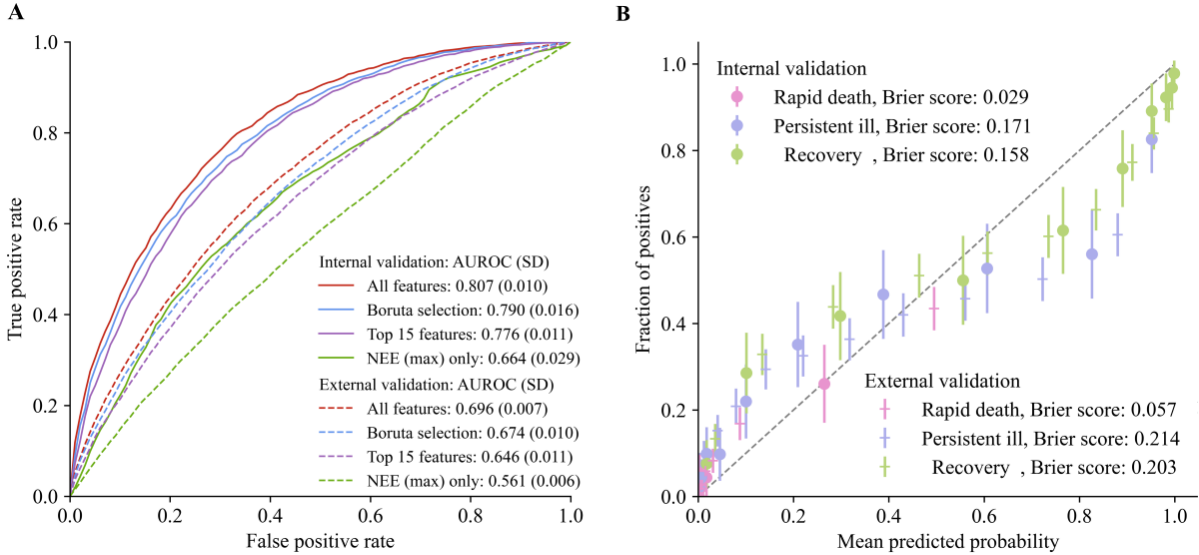
Performance results of the 3-way multiclass gradient boosting machine model. (A) Receiver operating characteristic curves for the model across different feature subsets in predicting persistent ill. (B) Calibration plots of each outcome for the model using all the features. The predicted probabilities were binned into deciles, and the mean and 95% CI were shown in each decile. AUROC: area under the receiver operating characteristic curve; NEE: norepinephrine equivalence.

When evaluating the discrimination performance of the multiclass GBM under 4 feature subsets in identifying persistent ill patients, on the internal validation cohort, the results showed that the model achieved AUROCs of 0.664-0.807 from using NEE (max) only to using all 148 features (Figure 4A). When applied to the external validation data set, the results were AUROCs of 0.561-0.696. Figure 4B shows the calibration plot. The full metric results in different feature subsets of all 3 trajectories are presented in Multimedia Appendix 8.

### Model Explanation

**Figure 5:**
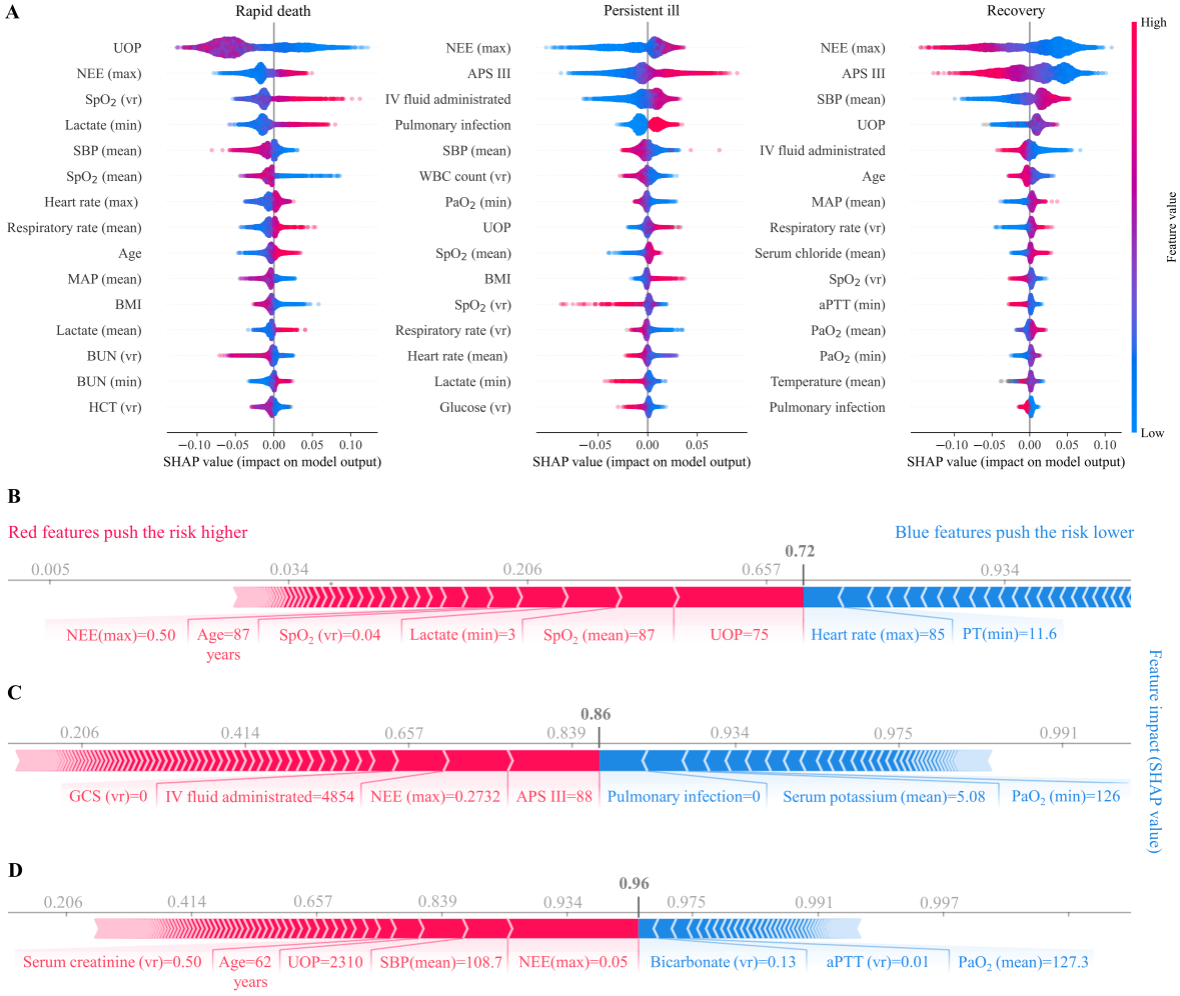
Model explanations. (A) Individual explanation summary of the top 15 clinical features for each trajectory. Beeswax plots show the feature importance across patients for the top 15 features, where each dot represents the feature importance value for 1 patient sample. Where multiple points fall on the same x position, they are stacked to show density. Features with positive impact values push risk up, while negative impact values push risk down. Long tails indicate features that are extremely important for some patients. Explanation of the output of the risk score for (B) a patient who did not survive, (C) a patient whose trajectory was persistent ill, and (D) a patient who recovered. APS: Acute Physiology Score; aPTT: activated partial thromboplastin time; BUN: blood urea nitrogen; GCS: Glasgow Coma Scale; HCT: hematocrit; IV: intravenous; MAP: mean arterial pressure; NEE: norepinephrine equivalence; PT: prothrombin time; SBP: systolic blood pressure; SHAP: Shapley Additive Explanations; SpO2: oxygen saturation; UOP: urine output; vr: variation; WBC: white blood cell.

The global feature importance for the multiclass GBM model using all the features is shown in Multimedia Appendix 9. The visible, individually interpretable summary of the impact of features across patients showed that oliguria, higher NEE (max), unstable oxygen saturation, higher lactate, and lower SBP were associated with a higher risk of rapid death; higher NEE (max), higher Acute Physiology Score III score, more IV fluid administrated, pulmonary infection, and lower SBP were associated with a higher risk of persistent illness. In contrast, patients with lower NEE (max), lower Acute Physiology Score III, higher SBP, more urine output, and less IV fluid administrated were more likely to recover (Figure 5A). For the example of explaining the individual prediction for different trajectories, see Figures 5B-5D.

### Conformal Prediction

**Figure 6:**
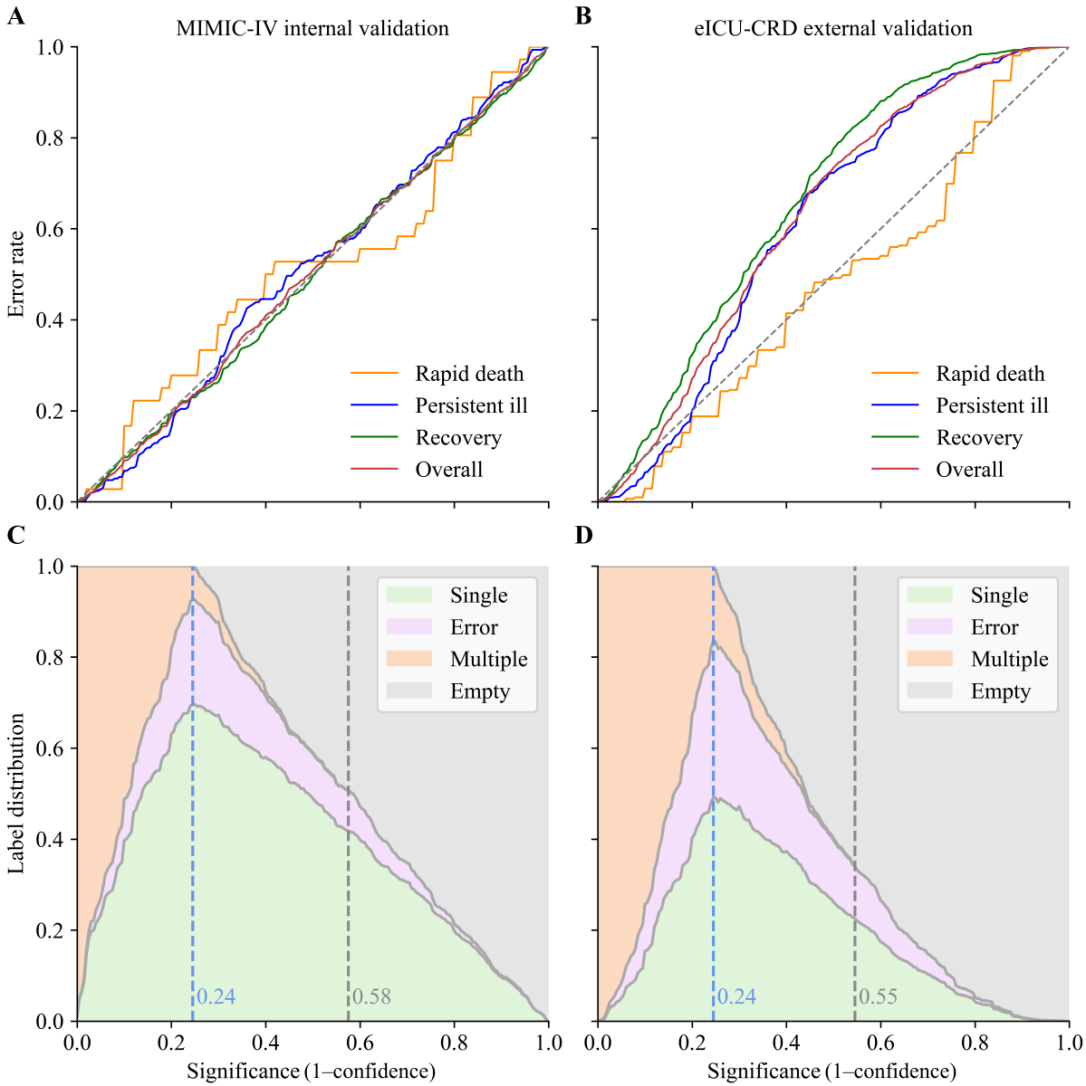
Plots of conformal prediction for the 2 validation cohorts. (A and B) The calibration curves depict the relationship between the observed prediction error (which represents the fraction of true labels not encompassed within the prediction region) on the y-axis and the prespecified significance level (the tolerated error rate) on the x-axis. The conformal predictor was well-calibrated for internal validation, while the calibration curve for external populations deviated from the ideal diagonal line but was relatively good for the persistent ill when the significance level was below 0.25. (C and D) Label distribution plots at different prespecified significance levels, with the model incorporating more multiple predictions at lower significance (higher confidence) levels. The blue dotted line indicates the corresponding significance level that produces the highest number of single-label predictions. The black dotted line indicates the corresponding significance level that first produces no multiple predictions. Single predictions output {rapid death} or {persistent ill} or {recovery}, multiple predictions output {rapid death, persistent ill}, {rapid death, recovery} or {persistent ill, recovery} or {rapid death, persistent ill, recovery}, and empty means {null}. eICU-CRD: eICU Collaborative Research Database; MIMIC-IV: Medical Information Mart for Intensive Care Database-IV.

The calibration curves of the observed prediction error were analyzed at different significance levels (1–confidence), ranging from 0% to 100% (Figure 6). When the model was evaluated using a mixed confidence approach, with a confidence level of 85% for recovery and a confidence level of 75% for rapid death and persistent ill, the model with CP reduced overall prediction errors by 27.6% (n=62) and 30.7% (n=412) in the internal and external validation cohorts, respectively, compared with the model without CP (Table 2). Specifically for the predictions in persistent ill patients, it made 69 (23.5%) errors in the internal validation cohort (compared with n=122, 41.5% errors without CP). In the external validation, it still produced significantly lower error rates compared with the model without CP (n=398, 31% vs n=689, 53.7%).

**Table 2:** Prediction regions on the internal validation and external validation cohort.

Prediction region			Internal validation cohort									External validation cohort						
			Rapid death (n=36), n (%)		Persistent ill (n=294), n (%)		Recovery (n=584), n (%)		Overall (n=914), n (%)		Rapid death (n=309), n (%)			Persistent ill (n=1283), n (%)		Recovery (n=2151), n (%)		Overall (n=3743), n (%)
**Confidence level: 90%**																		
	Empty	0 (0)		0 (0)		0 (0)		0 (0)		0 (0)			0 (0)		0 (0)		0 (0)	
	Error	6 (16.7)		20 (6.8)		57 (9.8)		83 (9.1)		9 (2.9)			83 (6.5)		292 (13.6)		384 (10.3)	
	Single	3 (8.3)		48 (16.3)		312 (53.4)		363 (39.7)		12 (3.9)			28 (2.2)		537 (25)		577 (15.4)	
	Multiple	27 (75)		226 (76.9)		215 (36.8)		468 (51.2)		288 (93.2)			1172 (91.3)		1322 (61.5)		2782 (74.3)	
**Confidence level: 85%**																		
	Empty	0 (0)		0 (0)		0 (0)		0 (0)		0 (0)			0 (0)		0 (0)		0 (0)	
	Error	8 (22.2)		35 (11.9)		84 (14.4)		127 (13.9)		34 (11)			163 (12.7)		474 (22)		671 (17.9)	
	Single	4 (11.1)		107 (36.4)		369 (63.2)		480 (52.5)		31 (10)			184 (14.3)		853 (39.7)		1068 (28.5)	
	Multiple	24 (66.7)		152 (51.7)		131 (22.4)		307 (33.6)		244 (79)			936 (73)		824 (38.3)		2004 (53.5)	
**Confidence level: 80%**																		
	Empty	0 (0)		0 (0)		0 (0)		0 (0)		0 (0)			0 (0)		0 (0)		0 (0)	
	Error	10 (27.8)		48 (16.3)		114 (19.5)		172 (18.8)		58 (18.8)			259 (20.2)		696 (32.4)		1013 (27.1)	
	Single	7 (19.4)		160 (54.4)		410 (70.2)		577 (63.1)		53 (17.2)			402 (31.3)		1053 (49)		1508 (40.3)	
	Multiple	19 (52.8)		86 (29.3)		60 (10.3)		165 (18.1)		198 (64.1)			622 (48.5)		402 (18.7)		1222 (32.6)	
**Confidence level: 75%**																		
	Empty	0 (0)		1 (0.3)		2 (0.3)		3 (0.3)		1 (0.3)			11 (0.9)		16 (0.7)		28 (0.7)	
	Error	10 (27.8)		68 (23.1)		132 (22.6)		210 (23)		57 (18.4)			387 (30.2)		839 (39)		1283 (34.3)	
	Single	10 (27.8)		178 (60.5)		448 (76.7)		636 (69.6)		85 (27.5)			488 (38)		1263 (58.7)		1836 (49.1)	
	Multiple	16 (44.4)		47 (16)		2 (0.3)		65 (7.1)		166 (53.7)			397 (30.9)		33 (1.5)		596 (15.9)	
**Mixed confidence level^a^**																		
	Empty	0 (0)		0 (0)		0 (0)		0 (0)		0 (0)			0 (0)		0 (0)		0 (0)	
	Error	10 (27.8)		69 (23.4)		84 (14.4)		163 (17.8)		58 (18.8)			398 (31)		474 (22)		930 (24.8)	
	Single	9 (25)		134 (45.6)		450 (77)		593 (64.9)		76 (24.6)			294 (22.9)		1279 (59.5)		1649 (44.1)	
	Multiple	17 (47.2)		91 (31)		50 (8.6)		158 (17.3)		175 (56.6)			591 (46.1)		398 (18.5)		1164 (31.1)	
**AI^b^ point prediction**																		
	Error	22 (61.1)		122 (41.5)		81 (13.9)		225 (24.6)		198 (64.1)			689 (53.7)		455 (21.2)		1342 (35.9)	
	Correct	14 (38.9)		172 (58.5)		503 (86.1)		689 (75.4)		111 (35.9)			594 (46.3)		1696 (78.8)		2401 (64.1)	

^a^85% for recovery, 75% for rapid death and persistent ill. The model demonstrates relatively improved performance in recognizing recovery due to a number of training examples for this class. As a result, when applying the conformal predictor, we can assign higher confidence levels within this class without generating excessively broad and uninformative prediction intervals.

^b^AI: artificial intelligence.

Although the number of accurately predicted persistent ill cases (efficiency) was lower compared to the conventional model without CP (n=134, 45.6% vs n=172, 58.5% in the internal cohort and n=294, 22.9% vs n=594, 46.3% in the external cohort), focusing on patients with multiple predictions of {rapid death, persistent ill} proved beneficial. Because among these predictions, 46 of 62 (74.2%) predictions were persistent ill in the internal validation cohort, and 351 of 496 (70.8%) predictions were persistent ill in the external validation cohort (Table 3).

**Table 3:** Multiple prediction analysis when set the mixed confidence level.

Multiple prediction^a^	Internal validation cohort					External validation cohort			
	Rapid death (n=17), n (%)	Persistent ill (n=91), n (%)	Recovery (n=50), n (%)	Total (n=158), n (%)	Rapid death (n=175), n (%)		Persistent ill (n=591), n (%)	Recovery (n=398), n (%)	Total (n=1164), n (%)
{0, 1}	16 (25.8)	46 (74.2)	—^b^	62 (39.2)	145 (29.2)		351 (70.8)	—	496 (42.6)
{0, 2}	1 (33.3)	—	2 (66.7)	3 (1.9)	15 (23.1)		—	50 (76.9)	65 (5.6)
{1, 2}	—	44 (48.9)	46 (51.1)	90 (57)	—		194 (40)	291 (60)	485 (41.7)
{0, 1, 2}	0 (0)	1 (33.3)	2 (66.7)	3 (1.9)	15 (12.7)		46 (39)	57 (48.3)	118 (10.1)

^a^Multiple prediction means that the prediction is uncertain, and the model could not distinguish between the possible class labels, thus awaiting the human review. Label 0 means rapid death, label 1 means persistent ill, and label 2 means recovery.

^b^Not applicable.

**Figure 7:**
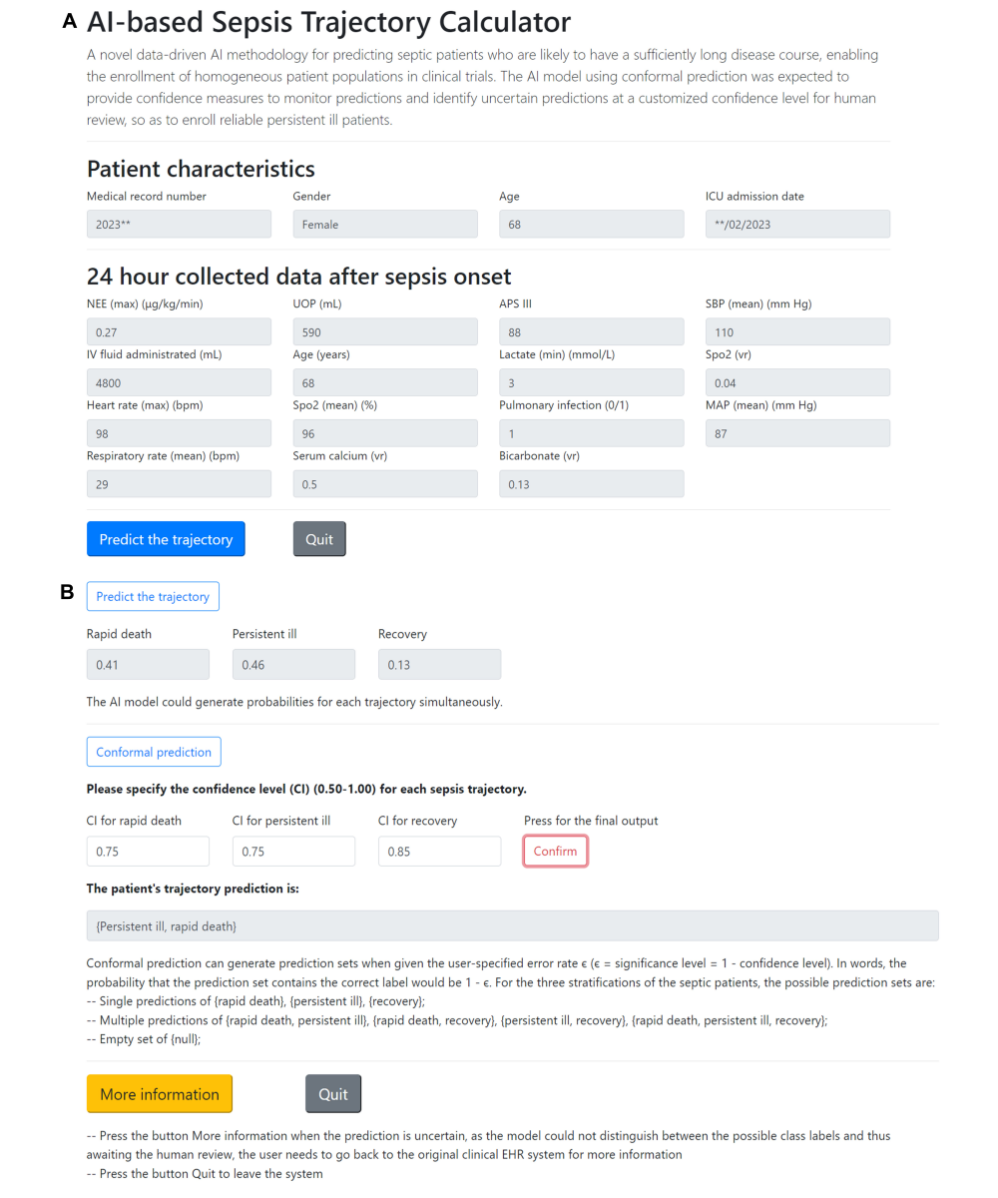
User interface of the web-based calculator. (A) Users can view the characteristics of the selected patient with sepsis and 24-hour collected feature values. (B) Generate risk scores for each sepsis trajectory by pressing the “Predict the trajectory” button. Then, press the “Conformal prediction” button, enter the confidence level for each sepsis trajectory, and press the “Confirm” button to output the final prediction under the conformal prediction framework. If the result was a multiple prediction, the users could press the “More information” button to go back to the original hospital electronic health record system to get more information for making a further decision, as the multiple prediction means that the prediction is uncertain as the model could not distinguish between the possible class labels and thus await human review. Users could also press “Quit” to leave the system. AI: artificial intelligence; APS: Acute Physiology Score; IV: intravenous; MAP: mean arterial pressure; NEE: norepinephrine equivalence; SBP: systolic blood pressure; SpO2: oxygen saturation; UOP: urine output.

Additionally, a web calculator was developed to enable users to visually understand the outputs of our AI model (Figure 7). This web-based calculator is internally deployed and is currently being updated at the Department of Critical Care Medicine, Zhongda Hospital. The web calculator is only accessible using a private URL by clinicians within the hospital and is connecting to the hospital’s EHR system. After a patient is identified as having sepsis and successfully collects 24-hour data after sepsis onset, the calculator would include this patient in the list. The users can then select 1 patient and view the relevant features that are calculated automatically based on data sourced from the hospital’s EHR system. After that, users can view the risk score predicted by this calculator and enter the confidence level to get a reliable prediction. The confidence level could be adjusted according to the model performance and workload, as a number of uncertain predictions can lead to an unmanageable situation.

## Discussion

### Principal Findings

We developed a novel AI-based model to predict patients with sepsis likely to have a long disease course. Such a model improves the homogeneity of patients with sepsis enrolled in clinical trials and ensures that a patient recruited into a trial will still be persistently ill by the time the proposed therapy can impact patient outcome. Furthermore, we used CP to estimate the uncertainty of the model outputs, which allows for a more comprehensive understanding of the model’s reliability and assists in making informed decisions based on the predicted outcomes. In addition, the developed web-based calculator may assist clinicians in comprehending the relationship between input data and predicted output. This could facilitate feedback from the clinicians and help improve the model predictions, which may be more effectively translated into medical practice for clinical decision-making.

To identify innovative pharmacological treatments for sepsis, enrolling patients who are most likely to benefit is essential. The enrichment strategy in this study differed from the prior prognostic and predictive enrichment strategies. In the Efficacy of Xuebijing Injection in Patients With Sepsis RCT, considering that the mortality rate was 74.3% in patients with a SOFA score of more than 13 in the Chinese population, Liu et al [25] used a prognostic enrichment strategy to enroll patients with sepsis with a SOFA score of 2 to 13 to evaluate the treatment effect of Xuebijing injection on 28-day mortality. However, this approach fails to consider patients with rapid recovery trajectories who may not derive significant benefits from Xuebijing injection treatment. Whether the targeted SOFA score can be effectively generalized in other trials remains uncertain. One important constraint with the predictive enrichment strategy is that different therapeutics necessitate different populations [26,27], and we lack the prior knowledge to accurately determine which patients are most likely to benefit from a specific therapy. Moreover, the limited availability of point-of-care devices for the identification of targeted patients, coupled with the lengthy measurement turnaround time, creates considerable obstacles for clinical trial enrichment [28]. These challenges are further exacerbated by the rapidly changing nature of diseases observed within clinical trials. From another perspective, we stratified patients based on the disease course of sepsis and suggested that future sepsis clinical trials should prioritize including patients with persistent ill, who would have a sufficiently long disease course to benefit from a therapy. Additionally, the current enrichment strategy does not necessarily take into account the different infection sites, pathogens, and pharmacological interventions.

Using real-time eHealth data, we developed an AI-based model for patient enrollment in sepsis clinical trials. Of the 12 machine learning models tested, a multiclass GBM model performed best and was able to provide a probability risk for each trajectory. The lightweight model using the top 15 features also showed acceptable performance and could be more suitable for future clinical applications. In addition, this model overcame the challenges of a heterogeneous patient population and tailored patient stratification to the needs of a particular drug candidate. Meanwhile, the predictive window, defined as the period between identification and enrollment, was 24 hours for the AI model, which was consistent with the previous sepsis RCTs [29]. It is reasonable to predict the disease course of patients within 24 hours after the identification of sepsis onset in future trials.

To mitigate the risk of automation bias in AI and evaluate model uncertainty, we implemented a CP framework on top of the prediction algorithm. This framework enabled the model to generate confidence measures, allowing for the monitoring of predictions and identification of uncertain outcomes based on adjustable confidence levels for human review. This is particularly crucial in different clinical scenarios where the AI system should be capable of acknowledging its limitations and saying “I don’t know” [30]. This could help to reduce prediction errors, especially in external validation, where the model performance could not be guaranteed, as well as in internal validation due to factors such as population heterogeneity and differences in clinical practice [31]. In addition, this study demonstrated that flagging the multiple (uncertain) predictions that the model could not classify for human review proved valuable, as it enabled the identification of more potentially persistent ill patients while simultaneously identifying high-risk individuals. In this case, the initial screening of patients could be achieved, allowing clinicians to avoid having to manually screen each patient for eligibility in the trial. Instead, they only need to review and assess the uncertain results of the model. As discussed by Goodman et al [32], clinicians were supposed to integrate their clinical experience with AI model outputs for clinical decision-making, thus avoiding inappropriate reliance on algorithms alone.

### Limitations

Our study faces some limitations. First, although this retrospective study demonstrates that the proposed AI-based enrichment strategy is effective in reducing patient heterogeneity in the design of sepsis clinical trials, we still need to include more patients to perform further prospective external validation using the developed web-based calculator. Second, the definition of sepsis onset was different between MIMIC-IV and eICU-CRD since the onset time of infection and organ failure in eICU-CRD were missing. Third, considering that our AI-based model needed a 24-hour predictive window after identifying sepsis onset, this model is not available for the mandated emergency therapies for sepsis, including the administration of antibiotics and IV fluid. The final limitation arises from the absence of criteria for balancing the trade-off between generating single-label and multiple predictions. Avoiding an excessive number of multiple (uncertain) predictions is important, as this can lead to an unmanageable situation.

### Conclusions

Our interpretable AI-based model using clinical data accurately identifies patients with different disease courses, which can reduce the heterogeneity of patients with sepsis in future clinical trials. Additionally, we estimate the uncertainty of the model outputs and enable the performance of the model using a mixed confidence approach. Application of this model can identify more homogeneous patient populations and precision pharmacological treatments for sepsis trials.

## Appendix

Multimedia Appendix 1 TRIPOD (Transparent Reporting of a Multivariable Prediction Model for Individual Prognosis or Diagnosis) checklist: prediction model development and validation.

Multimedia Appendix 2 Static and dynamic variables extracted and corresponding range.

Multimedia Appendix 3 Methods details.

Multimedia Appendix 4 Study population and exclusion criteria.

Multimedia Appendix 5 Top univariate associations with patient trajectory.

Multimedia Appendix 6 Model performance of the 12 machine learning model architecture and setup combinations.

Multimedia Appendix 7 Performance of the 3-way multiclass gradient boosting machine model using all the features for each outcome.

Multimedia Appendix 8 Results of the of multiclass gradient boosting machine in different feature selections in predicting different outcomes.

Multimedia Appendix 9 The overall global feature importance for the model using all the features.

## Abbreviations

AI: artificial intelligence
AUROC: area under the receiver operating characteristic curve
CP: conformal prediction
EHR: electronic health record
eICU-CRD: eICU Collaborative Research Database
GBM: gradient boosting machine
ICU: intensive care unit
IV: intravenous
LR: logistic regression
MIMIC-IV: Medical Information Mart for Intensive Care Database-IV
NDF: neural decision forest
NEE: norepinephrine equivalence
PPV: positive predictive value
RCT: randomized controlled trial
RF: random forest
SBP: systolic blood pressure
SOFA: Sequential Organ Failure Assessment
TPR: true positive rate
TRIPOD: Transparent Reporting of a Multivariable Prediction Model for Individual Prognosis or Diagnosis

## Data Availability

The data sets and code generated and analyzed during this study are available on the PhysioNet website [33,34] and GitHub repository [35], respectively.
