# Supplementary material for: Enhancing Patient Selection in Sepsis Clinical Trials Design Through an AI Enrichment Strategy: Algorithm Development and Validation
Source: J Med Internet Res. 2024 Sep 4;26:e54621. doi: 10.2196/54621 (PMC11411223; doi:10.2196/54621)
Supplement: Multimedia Appendix 2 [file jmir_v26i1e54621_app2.docx]

| **Variable name** | | **Name used in study** | **Limit** | **Unit** |
| --- | --- | --- | --- | --- |
| Static (9 variables) | | | | |
|  | Gender (male) | Gender (male) | 0/1 | 0/1 |
|  | Ethnicity (white) | Ethnicity (white) | 0/1 | 0/1 |
|  | Age | Age | 18-100 | years |
|  | Body mass index | BMI | 10-50 | kg/m² |
|  | Admission type (emergency) | Emergency admission | 0/1 | 0/1 |
|  | Pulmonary infection | Pulmonary infection | 0/1 | 0/1 |
|  | Invasive mechanical ventilation | Invasive MV | 0/1 | 0/1 |
|  | Renal replacement therapy first day | RRT | 0/1 | 0/1 |
|  | Acute physiology score III | APS III | 0-217 | n |
| Dynamic (37 variables) | | | | |
|  | Heart rate | Heart rate | 30-200 | beats/minute |
|  | Systolic blood pressure | SBP | 30-250 | mm Hg |
|  | Diastolic blood pressure | DBP | 30-150 | mm Hg |
|  | Mean arterial pressure | MAP | 30-150 | mm Hg |
|  | Respiratory rate | Respiratory rate | 1-60 | breaths/minute |
|  | Temperature | Temperature | 30-45 | ℃ |
|  | SpO_2_ | SpO_2_ | 30-100 | % |
|  | pH | pH | 6.0-8.0 | - |
|  | Lactate | Lactate | 0-10 | mmol/L |
|  | PaO_2_ | PaO_2_ | 30-500 | mm Hg |
|  | FiO_2_ | FiO_2_ | 21-100 | % |
|  | PaCO_2_ | PaCO_2_ | 10-150 | mm Hg |
|  | PaO_2_/FiO_2_ ratio | PaO_2_/FiO_2_ ratio | 30-650 | mm Hg |
|  | Base excess | Base excess | -30-30 | mmol/L |
|  | Alanine transaminase | ALT | 0-3000 | IU/L |
|  | Aspartate transferase | AST | 0-3000 | IU/L |
|  | Total bilirubin | Total bilirubin | 0-20 | mg/dL |
|  | Albumin | Albumin | 0-7 | g/dL |
|  | Bicarbonate | Bicarbonate | 5-50 | mmol/L |
|  | Blood urea nitrogen | BUN | 0-115 | mg/dL |
|  | Serum calcium | Serum calcium | 0-50 | mmol/L |
|  | Serum chloride | Serum chloride | 60-150 | mmol/L |
|  | Serum creatinine | Serum creatinine | 0-8 | mg/dL |
|  | Glucose | Glucose | 0-800 | mg/dL |
|  | Serum sodium | Serum sodium | 80-200 | mmol/L |
|  | Serum potassium | Serum potassium | 0-12 | mmol/L |
|  | International normalized ratio | INR | 0-15 | - |
|  | Prothrombin time | PT | 5-100 | seconds |
|  | Activated partial thromboplastin time | aPTT | 20-200 | seconds |
|  | Hematocrit | HCT | 10-55 | % |
|  | Hemoglobin | Hb | 0-20 | g/dL |
|  | Platelet count | PLT count | 0-1000 | K/μL |
|  | White blood cell count | WBC count | 0-60 | K/μL |
|  | Glasgow coma scale | GCS | 3-15 | n |
|  | Total urine output | UOP | 0-20000 | mL |
|  | Total intravenous fluid administrated | IV fluid administrated | 0-20000 | mL |
|  | Maximum norepinephrine equivalence | NEE (max) | 0-5.0 | μg/kg/min |
