# Supplementary material for: Enhancing Patient Selection in Sepsis Clinical Trials Design Through an AI Enrichment Strategy: Algorithm Development and Validation
Source: J Med Internet Res. 2024 Sep 4;26:e54621. doi: 10.2196/54621 (PMC11411223; doi:10.2196/54621)
Supplement: Multimedia Appendix 3 [file jmir_v26i1e54621_app3.docx]

## **1. Predictive algorithm**

(1) Gradient Boosting Machine

The eXtreme Gradient Boosting (XGBoost) [1] machine was used in this study. XGBoost is an optimized implementation of the gradient boosting framework, which combines multiple weak predictive models (typically decision trees) to create a strong predictive model. The key idea behind XGBoost is to iteratively build a series of decision trees, each of which attempts to correct the errors made by the previous trees in the ensemble. This iterative process is called boosting. The trees are built sequentially, with each subsequent tree focusing on the remaining errors of the ensemble up to that point. By continuously adding trees and refining the model, XGBoost improves its prediction accuracy with each iteration. It calculates the gradients of the loss function with respect to the predictions of the current ensemble, and then updates the model parameters in the direction that minimizes the loss. XGBoost can learn how to effectively handle missing values in the input features during training, eliminating the need for preprocessing steps such as imputation.

The package in Python ([https://xgboost.readthedocs.io/en/stable/index.html#](https://xgboost.readthedocs.io/en/stable/index.html)) was used for XGBoost.

(2) Neural Decision Forest

Deep Neural Decision Forest (NDF) [2] model was first developed for structured data classification. The NDF model showcases the creation of a differentiable decision tree model that is both stochastic and trainable end-to-end. It achieves this by integrating decision trees with deep representation learning. The model employs two sets of weights for learning: the first set represents the class probability distribution in the tree leaves, while the second set comprises the weights of the routing layer, indicating the probabilities of traversing each leaf. During the forward pass, it expects input features in the form of a single vector that encodes all the instance features within a batch. This vector can be generated by dense transformations applied to structured data features. The model randomly selects a subset of the input features, then iteratively computes the probabilities of the input instances reaching the tree leaves using stochastic routing through different tree levels. The probabilities of reaching the leaves are ultimately merged with the class probabilities present at the leaves in order to generate the outputs. Finally, the NDF model comprises a collection of neural decision trees that are trained concurrently. The output of NDF is determined by averaging the outputs of its individual trees.

We used the package (<https://keras.io/examples/structured_data/deep_neural_decision_forests/>) in Python to develop the NDF model.

(3) Random Forest

Random Forest (RF) is an ensemble learning algorithm that combines the predictions of multiple decision trees to achieve better overall performance. It is based on the theory of randomization and aggregation. During training, RF randomly selects subsets of features from the available feature set to decorrelate the trees and reduce overfitting. The algorithm also uses bagging, where multiple subsets of the training data are created by sampling with replacement. Each decision tree is trained independently on these subsets, and the final prediction is obtained by aggregating the individual tree predictions through classification voting. The key idea of RF is to exploit the wisdom of the crowd, where the combination of different decision trees helps to capture a wider range of patterns and improve prediction accuracy.

Python in <https://scikit-learn.org/stable/modules/generated/sklearn.ensemble.RandomForestClassifier.html> was implemented for constructing RF.

(4) Logistic Regression

Logistic Regression (LR) is a statistical modeling technique used for classification and predictive analytics. The theory behind LR is rooted in the logistic function, which maps input values to a probability range between 0 and 1. It models the relationship between the independent variables (features) and the dependent variable (binary outcome) using the logistic function. The goal of LR is to find the optimal coefficients that maximize the likelihood of observing the given data. The beta parameter, or coefficient is estimated using the maximum likelihood estimation method. During training, LR calculates the probabilities of the binary outcomes based on the feature values and iteratively adjusts the coefficients using optimization algorithms such as gradient descent.

Python in <https://scikit-learn.org/stable/modules/generated/sklearn.linear_model.LogisticRegression.html> was implemented for constructing LR.

## **2. Model explanation**

We used SHAP (SHapley Additive exPlanations) [3] method for explaining the machine learning models. SHAP values are from the game theory literature, providing a fair way to distribute the "credit" among the features by considering all possible combinations of features and measure their impact on the prediction. It provides an interpretable measure of feature importance by assigning a value to each feature for a particular prediction. For a machine learning model *f*, the SHAP value represented as $\phi_{i}\left( f,x \right)$, is a single numerical value representing the impact of feature *i* (such as age, lactate, systolic blood pressure, etc.) on the prediction of the model *f* when given the input patient data *x*.

$\phi_{i}\left( f,x \right)=\sum_{S\subseteq S_{all/\{i\}}} \frac{\left| S \right|!\left( M-\left| S \right|-1 \right)!}{M！}\left[ f_{x}\left( S\cup\left\{ i \right\} \right)-f_{x}\left( S \right) \right]$

$=\sum_{S\subseteq S_{all/\{i\}}} \frac{1}{(M\mathrm{choose}\left| S \right|)(M-\left| S \right|)}\left[ f_{x}\left( S\cup\left\{ i \right\} \right)-f_{x}\left( S \right) \right]$

Where *S* is the subset of the input features*, |S|* is the number of set elements*, M* is the number of features, *x_s_* represents the input under the feature vector subset, $f_{x}\left( S \right)=E[f(x)|x_{s}]$. The calculation method of the global importance $\psi_{i}$ of a feature *i* to the model in all *N* sample spaces *X* is as follows:

$$\psi_{i}=\frac{|\sum_{n=1}^{N} \phi_{i}\left( f,X_{n} \right)|}{N}$$

SHAP analysis was performed in Python ([https://shap.readthedocs.io/en/latest/index.html#](https://shap.readthedocs.io/en/latest/index.html)).

## **3. Feature subset**

(1) The 15 most important features

The features subset of the 15 most important was obtained with the model global feature importance measured by SHAP values.

(2) Boruta feature selection method

Boruta is a feature selection [4] method that aims to identify the most relevant features in a dataset. It is based on the concept of RF and uses a randomized approach to determine the importance of features. Boruta compares the importance of each feature with that of randomly generated shadow features created by permuting the values of a given feature. By comparing the importance of the original features with that of the shadow features, Boruta determines whether a feature is truly important or whether its importance is similar to random noise. The algorithm iteratively performs tests to assess the importance of features and classifies them as "confirmed", "tentative" or "rejected". Confirmed features are considered truly important, while rejected features are considered unimportant. Tentative features require further investigation.

We used Package in python (<https://github.com/scikit-learn-contrib/boruta_py>) for the Boruta feature selection.

(3) Using only the maximum norepinephrine equivalence

Model was retrained using only the maximum norepinephrine equivalence (NEE), *i.e.*, NEE (max).

## **4. Parameters Bayesian optimization**

The Bayesian optimization [5] method is a powerful technique used to optimize the hyperparameters of machine learning models. It combines the principles of Bayesian inference and optimization to efficiently search the hyperparameter space. The method constructs a probabilistic model, known as a surrogate model, to approximate the objective function and its uncertainty. By iteratively sampling and evaluating new hyperparameter configurations, the algorithm updates the surrogate model and determines the most promising regions of the search space. This iterative process guides the optimization towards the optimal set of hyperparameters, ultimately improving the performance of the machine learning model.

The Package in python (<http://hyperopt.github.io/hyperopt/>) was used for the parameters Bayesian optimization.

| **Model** | **Parameters** | **Search space^a^** |
| --- | --- | --- |
| Gradient Boosting Machine | max_depths | arange(2, 11, 1) |
|  | learning_rates | [0.001, 0.01, 0.02, 0.04, 0.06, 0.08, 0.1, 0.15, 0.2, 0.3] |
|  | subsamples | arange(0.5, 1.1, 0.1) |
|  | colsample_bytrees | arange(0.5, 1.1, 0.1) |
|  | reg_alphas | [0.0, 0.005, 0.01, 0.05, 0.1] |
|  | reg_lambdas | [0.8, 1, 1.5, 2, 4] |
| Neural Decision Forest | num_trees | arange(10, 100, 10) |
|  | depth | arange(2, 11, 1) |
|  | used_features_rate | arange(0.1, 1.1, 0.1) |
|  | learning_rate | [0.001, 0.005, 0.01] |
|  | batch_size | [32, 64, 128] |
|  | max_epochs | arange(50, 250, 50) |
| Random Forest | n_estimators | arange(100, 600, 100) |
|  | max_samples | arange(0.5, 1.1, 0.1) |
|  | min_samples_leaf | arange(0.5, 1.1, 0.1) |
| Logistic Regression | solvers | ['newton-cg', 'sag', 'saga', 'lbfgs'] |
|  | C | arange(0.1, 1.1, 0.1) |
|  | max_iter | arange(50, 150, 10) |

**^a^**arange(minimum, maximum, step), for example, arange(0, 5, 1)=[0, 1, 2, 3, 4]

## **5. Conformal prediction**

Conformal prediction (CP) is a method that can be mathematically guaranteed to produce statistical uncertainty sets/intervals for unknown samples that differ from the training data [6]. When selecting the multiclass predicted model *f* that outputs estimated probabilities (softmax scores) for each class (e.g. {rapid death, persistent ill, recovery}), CP can generate prediction sets when given a user-specified error rate є (є = significance level = 1 - confidence level) using additional calibration data^7^. In words, the probability that the prediction set contains the correct label would be 1 - є. Here are the following steps to develop the conformal predictor.

**Step 1: Calculate the nonconformity score.** Central to CP is the use of nonconformity measures to assess how dissimilar a new sample is from the data on which the model was built. In this study, we used a commonly used nonconformity measure, which is the predicted probability of an example belonging to a specific class, to determine the nonconformity score. By using this nonconformity measure and a dataset, we can calculate the nonconformity score $s_{i} (i=1,\ldots,N)$ for each labeled example $x_{i}$. Subsequently, a test example $x_{N+1}$ will be assigned a potential label (e.g. {persistent ill}). To identify the most fitting label based on the previous data, we evaluate s for every possible potential label for the example.

**Step 2: Compute the** *P* **values.** In order to assess the conformity of a potential label for a test example $x_{N+1}$ with the existing data, we count the number of $s_{i} (i=1,\ldots,N)$that are equal to or greater than the $s_{N+1}$ of the test example. This count is then divided by N+1. This resulting ratio represents the proportion of training examples that are at least as conforming as the test example, known as the *P* value. A higher *P* value indicates a greater level of confidence that the assigned label aligns the example with the previous data. The *P* values are used slightly differently than in standard hypothesis testing in statistics. In essence, these *P* values are the ranking of a test object compared to known instances of each class. The Mondrian conformal predictor [6] was specifically used in this research for outputting *P* values for each class. It could work on a class basis to ensure the desired error rate within each class.

**Step 3: Generate prediction sets for new samples.** The prediction sets are calculated from these *P* values together with the desired confidence by finding the *P* values that are equal to or larger than the significance threshold є = 1 - confidence level, *i.e.*, the percentage of accepted errors. By only including *P* values over the desired confidence level, the produced prediction sets contatins the true label with a probability of 1 - є. In this study, the possible prediction sets are: single predictions of {0}, {1}, {2}; multiple predictions of {0, 1}, {0, 2}, {1, 2}, {0, 1, 2}; empty set of {null}, where label 0 means rapid death, label 1 means persistent ill and label 2 means recovery. For multiple predictions, it means that the prediction was uncertain, and the model could not distinguish between the possible class labels, so new information should be provided in order to result in a single class prediction. Empty set predictions were examples where the model could not assign any label. An example of how the conformal prediction was made at different significance levels is shown below.

**
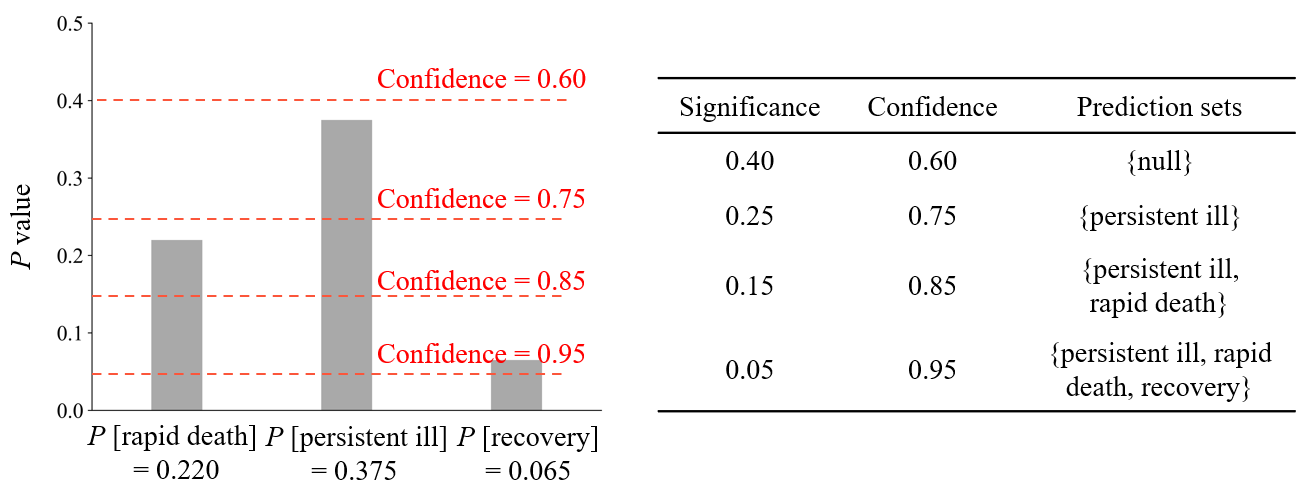
**

With a higher confidence level, we get fewer error predictions but more multiple predictions, *i.e.*, we can not make a single-class prediction, and ask more for human review. An example is shown in the table below. Therefore, we should consider the trade-off between producing single and multiple predictions according to the clinical need.

Package (<https://search.r-project.org/CRAN/refmans/conformalClassification/html/conformalClassification.html>) in R studio was used for the CP.

| **Patient** | **Confidence level = 80%** | | |  | **Confidence level = 90%** | | |
| --- | --- | --- | --- | --- | --- | --- | --- |
|  | **True label^a^** | **Predicted sets^a^** | **Prediction regions** |  | **True label^a^** | **Predicted sets^a^** | **Prediction regions** |
| 1 | 1 | {0, 1} | Multiple |  | 1 | {0, 1} | Multiple |
| 2 | 2 | {0, 1} | Error |  | 2 | {0, 1, 2} | Multiple |
| 3 | 0 | {0} | Single |  | 0 | {0} | Single |
| 4 | 1 | {1} | Single |  | 1 | {0, 1} | Multiple |
| 5 | 2 | {2} | Single |  | 2 | {1, 2} | Multiple |
| 6 | 1 | {2} | Error |  | 1 | {1, 2} | Multiple |
| 7 | 0 | {0} | Single |  | 0 | {0} | Single |
| 8 | 1 | {1} | Single |  | 1 | {1} | Single |
| 9 | 1 | {null} | Empty |  | 1 | {0, 1} | Multiple |
| 10 | 2 | {1} | Error |  | 2 | {1} | Error |
| 11 | 0 | {0, 1} | Multiple |  | 0 | {0, 1} | Multiple |
| 12 | 1 | {2} | Error |  | 1 | {2} | Error |

^a^ where label 0 means rapid death, label 1 means persistent ill and label 2 means recovery

**Reference**

1. Chen T, Guestrin C. XGBoost: A scalable tree boosting system. Proceedings of the 22nd ACM SIGKDD International Conference on Knowledge Discovery and Data Mining. San Francisco, California, USA: Association for Computing Machinery; 2016. p. 785-94.

2. Kontschieder P, Fiterau M, Criminisi A, Bulò SR. Deep neural decision forests. 2015 IEEE International Conference on Computer Vision (ICCV); 2015. p. 1467-75.

3. Lundberg SM, Erion G, Chen H, et al. From local explanations to global understanding with explainable AI for trees. *Nat Mach Intell* 2020; 2(1): 56-67.

4. Kursa MB, Rudnicki WR. Feature selection with the boruta package. *Journal of Statistical Software* 2010; 36(11): 1-13.

5. Shahriari B, Swersky K, Wang Z, Adams RP, Freitas Nd. Taking the human out of the loop: A review of bayesian optimization. *Proceedings of the IEEE* 2016; 104(1): 148-75.

6. Alvarsson J, McShane SA, Norinder U, Spjuth O. Predicting with confidence: Using conformal prediction in drug discovery. *J Pharm Sci-Us* 2021; 110(1): 42-9.

7. Olsson H, Kartasalo K, Mulliqi N, et al. Estimating diagnostic uncertainty in artificial intelligence assisted pathology using conformal prediction. *Nat Commun* 2022; 13(1): 7761.
