# Supplementary figures and images for: Enhancing Patient Selection in Sepsis Clinical Trials Design Through an AI Enrichment Strategy: Algorithm Development and Validation
Source: J Med Internet Res. 2024 Sep 4;26:e54621. doi: 10.2196/54621 (PMC11411223; doi:10.2196/54621)

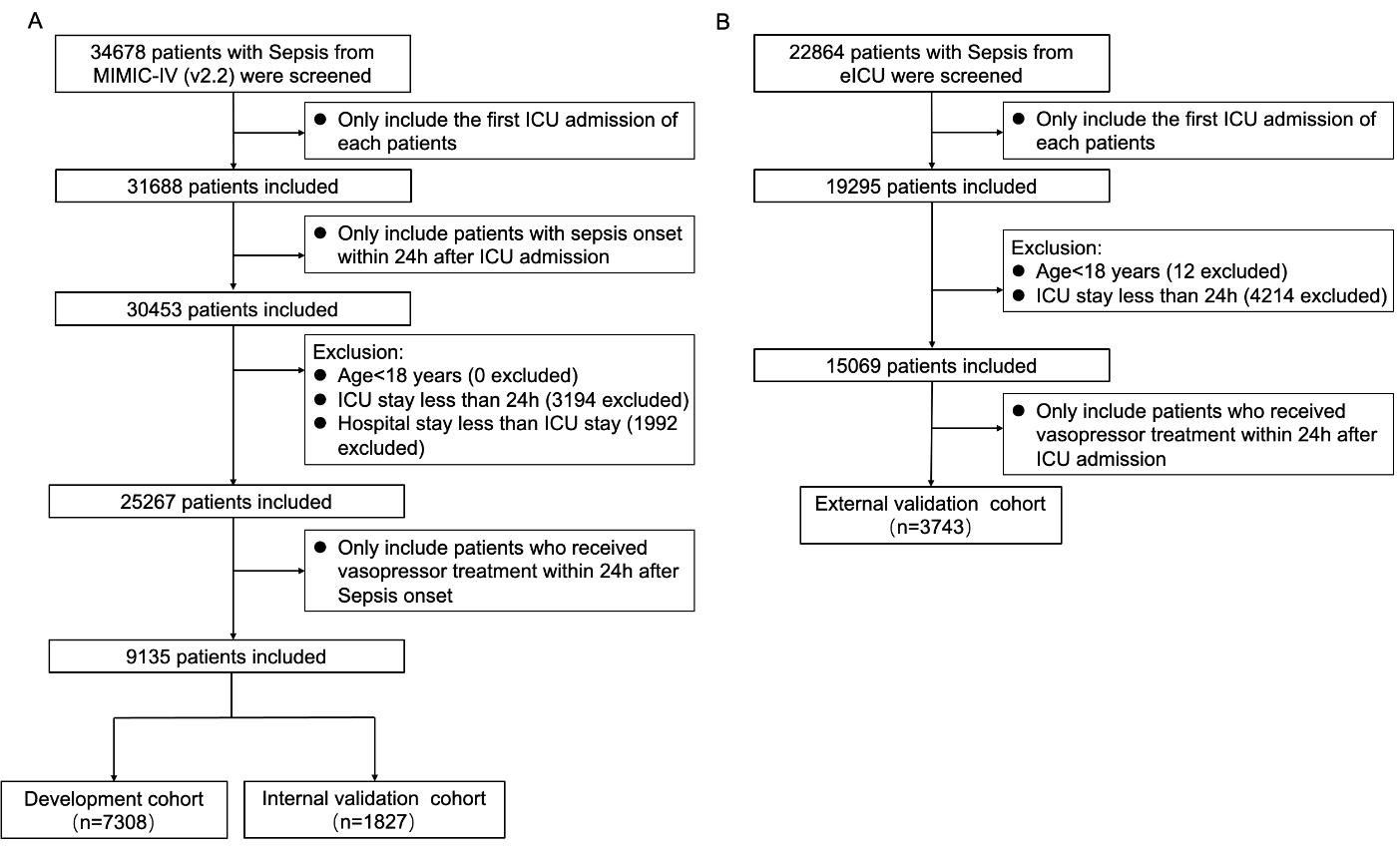

Supplement: Multimedia Appendix 4 [file jmir_v26i1e54621_app4.docx]
