# Supplementary material for: Enhancing Patient Selection in Sepsis Clinical Trials Design Through an AI Enrichment Strategy: Algorithm Development and Validation
Source: J Med Internet Res. 2024 Sep 4;26:e54621. doi: 10.2196/54621 (PMC11411223; doi:10.2196/54621)
Supplement: Multimedia Appendix 5 [file jmir_v26i1e54621_app5.docx]

| **Rank** | | **Feature** | | **Odds ratio^a^** |  | **Feature** | **Odds ratio^b^** | |
| --- | --- | --- | --- | --- | --- | --- | --- | --- |
| **Rapid death** | | |  | |  |  | |  |
| 1 | APS III | | | 2.44 (2.22-2.68) |  | UOP | 0.22 (0.18-0.27) | |
| 2 | Respiratory rate (mean) | | | 2.04 (1.87-2.24) |  | Bicarbonate (mean) | 0.35 (0.32-0.40) | |
| 3 | Lactate (mean) | | | 2.00 (1.87-2.14) |  | Bicarbonate (min) | 0.36 (0.32-0.40) | |
| 4 | Lactate (max) | | | 1.94 (1.82-2.08) |  | Base excess (mean) | 0.40 (0.37-0.44) | |
| 5 | Lactate (min) | | | 1.93 (1.81-2.06) |  | Bicarbonate (max) | 0.43 (0.38-0.48) | |
| 6 | Heart rate (mean) | | | 1.78 (1.62-1.96) |  | pH (mean) | 0.44 (0.40-0.48) | |
| 7 | Bicarbonate (vr) | | | 1.70 (1.59-1.82) |  | Base excess (min) | 0.44 (0.41-0.48) | |
| 8 | Serum potassium (mean) | | | 1.69 (1.54-1.86) |  | Base excess (max) | 0.47 (0.43-0.51) | |
| 9 | Heart rate (max) | | | 1.68 (1.54-1.84) |  | SBP (mean) | 0.49 (0.43-0.55) | |
| 10 | IV fluid administrated | | | 1.67 (1.56-1.79) |  | SBP (min) | 0.49 (0.45-0.54) | |
| **Persistent ill** | | | | | | | | |
| 1 | APS III | | | 2.26 (2.15-2.38) |  | PaO_2_ (mean) | 0.61 (0.58-0.64) | |
| 2 | IV fluid administrated | | | 1.64 (1.56-1.72) |  | PaO_2_ (min) | 0.70 (0.66-0.75) | |
| 3 | NEE (max) | | | 1.63 (1.52-1.75) |  | Bicarbonate (min) | 0.71 (0.68-0.74) | |
| 4 | Pulmonary infection | | | 1.44 (1.38-1.50) |  | PaO_2_ (max) | 0.72 (0.69-0.75) | |
| 5 | BUN (max) | | | 1.39 (1.34-1.46) |  | PaO_2_/FiO_2_ ratio (min) | 0.74 (0.70-0.77) | |
| 6 | Invasive MV | | | 1.39 (1.32-1.45) |  | SBP (mean) | 0.74 (0.71-0.78) | |
| 7 | Serum creatinine (max) | | | 1.38 (1.32-1.44) |  | Base excess (min) | 0.75 (0.72-0.78) | |
| 8 | BUN (mean) | | | 1.37 (1.31-1.43) |  | Bicarbonate (mean) | 0.76 (0.73-0.80) | |
| 9 | Serum creatinine (mean) | | | 1.35 (1.30-1.41) |  | Base excess (mean) | 0.76 (0.73-0.80) | |
| 10 | BUN (min) | | | 1.33 (1.28-1.39) |  | PaO_2_/FiO_2_ ratio (mean) | 0.76 (0.73-0.80) | |
| **Recovery** | | | | | | | | |
| 1 | PaO_2_ (mean) | | | 1.77 (1.68-1.86) |  | NEE (max) | 0.34 (0.31-0.37) | |
| 2 | Bicarbonate (min) | | | 1.70 (1.62-1.78) |  | APS III | 0.35 (0.33-0.37) | |
| 3 | Base excess (min) | | | 1.62 (1.54-1.69) |  | IV fluid administrated | 0.50 (0.48-0.53) | |
| 4 | Base excess (mean) | | | 1.61 (1.54-1.69) |  | Lactate (mean) | 0.57 (0.54-0.60) | |
| 5 | Bicarbonate (mean) | | | 1.57 (1.49-1.64) |  | Lactate (min) | 0.57 (0.54-0.60) | |
| 6 | pH (mean) | | | 1.54 (1.47-1.61) |  | Lactate (max) | 0.62 (0.59-0.65) | |
| 7 | pH (min) | | | 1.53 (1.46-1.60) |  | BUN (max) | 0.65 (0.62-0.68) | |
| 8 | PaO_2_ (min) | | | 1.50 (1.41-1.59) |  | Respiratory rate (mean) | 0.66 (0.63-0.69) | |
| 9 | SBP (mean) | | | 1.49 (1.42-1.56) |  | BUN (mean) | 0.66 (0.63-0.69) | |
| 10 | PaO_2_ (max) | | | 1.47 (1.41-1.54) |  | Serum creatinine (max) | 0.66 (0.63-0.69) | |

Top univariate associations with patient trajectory. Univariate logistic regressions were run associating each variable with three trajectories: (1) rapid death vs. recovery or persistent ill; (2) persistent ill vs. recovery or rapid death; (3) recovery vs. rapid death or persistent ill.

^a^ Sort the odds ratio values from highest to lowest.

^b^ Sort the odds ratio values from lowest to highest.
