# Supplementary material for: Enhancing Patient Selection in Sepsis Clinical Trials Design Through an AI Enrichment Strategy: Algorithm Development and Validation
Source: J Med Internet Res. 2024 Sep 4;26:e54621. doi: 10.2196/54621 (PMC11411223; doi:10.2196/54621)
Supplement: Multimedia Appendix 6 [file jmir_v26i1e54621_app6.docx]

| **Set up** | **Model** | **Metric**  **mean (SD)** | **MIMIC-IV internal validation** | | |  | **eICU-CRD external validation** | | |
| --- | --- | --- | --- | --- | --- | --- | --- | --- | --- |
|  |  |  | **Rapid death** | **Persistent ill** | **Recovery** |  | **Rapid death** | **Persistent ill** | **Recovery** |
| Multiclass | Gradient boosting machine | AUROC | 0.906 (0.018) | 0.807 (0.010) | 0.843 (0.008) |  | 0.878 (0.003) | 0.696 (0.007) | 0.764 (0.008) |
|  |  | AUPRC | 0.462 (0.038) | 0.656 (0.019) | 0.901 (0.007) |  | 0.481 (0.009) | 0.531 (0.006) | 0.803 (0.010) |
|  |  | F-0.5 | 0.494 (0.038) | 0.611 (0.015) | 0.813 (0.009) |  | 0.518 (0.017) | 0.511 (0.009) | 0.726 (0.007) |
|  |  | PPV | 0.608 (0.082) | 0.618 (0.017) | 0.805 (0.013) |  | 0.612 (0.040) | 0.522 (0.010) | 0.711 (0.012) |
|  |  | TPR | 0.306 (0.073) | 0.589 (0.036) | 0.848 (0.020) |  | 0.331 (0.054) | 0.478 (0.044) | 0.792 (0.027) |
|  | Random forest | AUROC | 0.905 (0.017) | 0.791 (0.013) | 0.829 (0.012) |  | 0.878 (0.001) | 0.679 (0.003) | 0.772 (0.001) |
|  |  | AUPRC | 0.411 (0.071) | 0.631 (0.022) | 0.890 (0.009) |  | 0.469 (0.007) | 0.480 (0.005) | 0.808 (0.001) |
|  |  | F-0.5 | 0.241 (0.149) | 0.584 (0.025) | 0.780 (0.008) |  | 0.008 (0.008) | 0.452 (0.007) | 0.708 (0.002) |
|  |  | PPV | 0.626 (0.363) | 0.654 (0.028) | 0.750 (0.009) |  | 0.450 (0.472) | 0.505 (0.007) | 0.670 (0.003) |
|  |  | TPR | 0.074 (0.050) | 0.411 (0.029) | 0.931 (0.010) |  | 0.002 (0.002) | 0.319 (0.010) | 0.913 (0.005) |
|  | Neural decision forest | AUROC | 0.899 (0.024) | 0.779 (0.013) | 0.832 (0.011) |  | 0.861 (0.006) | 0.651 (0.008) | 0.758 (0.003) |
|  |  | AUPRC | 0.482 (0.035) | 0.617 (0.018) | 0.892 (0.009) |  | 0.449 (0.011) | 0.474 (0.009) | 0.796 (0.002) |
|  |  | F-0.5 | 0.397 (0.033) | 0.576 (0.017) | 0.820 (0.008) |  | 0.404 (0.020) | 0.485 (0.009) | 0.728 (0.004) |
|  |  | PPV | 0.374 (0.032) | 0.564 (0.017) | 0.835 (0.009) |  | 0.378 (0.026) | 0.478 (0.009) | 0.749 (0.014) |
|  |  | TPR | 0.526 (0.044) | 0.629 (0.023) | 0.765 (0.015) |  | 0.560 (0.038) | 0.519 (0.057) | 0.656 (0.037) |
|  | Logistic regression | AUROC | 0.868 (0.027) | 0.719 (0.031) | 0.813 (0.010) |  | 0.764 (0.039) | 0.505 (0.032) | 0.692 (0.017) |
|  |  | AUPRC | 0.401 (0.056) | 0.556 (0.029) | 0.874 (0.010) |  | 0.265 (0.046) | 0.368 (0.022) | 0.718 (0.019) |
|  |  | F-0.5 | 0.241 (0.029) | 0.546 (0.024) | 0.812 (0.007) |  | 0.190 (0.034) | 0.323 (0.054) | 0.678 (0.014) |
|  |  | PPV | 0.209 (0.025) | 0.549 (0.025) | 0.834 (0.011) |  | 0.164 (0.039) | 0.409 (0.029) | 0.723 (0.018) |
|  |  | TPR | 0.639 (0.082) | 0.536 (0.041) | 0.735 (0.021) |  | 0.716 (0.166) | 0.209 (0.117) | 0.547 (0.064) |
| Two-way | Gradient boosting machine | AUROC | - | 0.806 (0.010) | - |  | - | 0.689 (0.007) | - |
|  |  | AUPRC | - | 0.653 (0.024) | - |  | - | 0.522 (0.012) | - |
|  |  | F-0.5 | - | 0.627 (0.016) | - |  | - | 0.513 (0.009) | - |
|  |  | PPV | - | 0.666 (0.051) | - |  | - | 0.525 (0.019) | - |
|  |  | TPR | - | 0.534 (0.094) | - |  | - | 0.478 (0.050) | - |
|  | Random forest | AUROC | - | 0.795 (0.010) | - |  | - | 0.699 (0.003) | - |
|  |  | AUPRC | - | 0.633 (0.018) | - |  | - | 0.524 (0.004) | - |
|  |  | F-0.5 | - | 0.610 (0.018) | - |  | - | 0.518 (0.005) | - |
|  |  | PPV | - | 0.631 (0.041) | - |  | - | 0.522 (0.018) | - |
|  |  | TPR | - | 0.561 (0.092) | - |  | - | 0.513 (0.065) | - |
|  | Neural decision forest | AUROC | - | 0.796 (0.011) | - |  | - | 0.664 (0.006) | - |
|  |  | AUPRC | - | 0.639 (0.021) | - |  | - | 0.486 (0.007) | - |
|  |  | F-0.5 | - | 0.620 (0.016) | - |  | - | 0.485 (0.008) | - |
|  |  | PPV | - | 0.652 (0.038) | - |  | - | 0.480 (0.018) | - |
|  |  | TPR | - | 0.532 (0.069) | - |  | - | 0.519 (0.072) | - |
|  | Logistic regression | AUROC | - | 0.780 (0.010) | - |  | - | 0.579 (0.013) | - |
|  |  | AUPRC | - | 0.612 (0.017) | - |  | - | 0.422 (0.017) | - |
|  |  | F-0.5 | - | 0.607 (0.014) | - |  | - | 0.428 (0.009) | - |
|  |  | PPV | - | 0.621 (0.040) | - |  | - | 0.433 (0.033) | - |
|  |  | TPR | - | 0.581 (0.098) | - |  | - | 0.434 (0.075) | - |
| Three-way nested | Gradient boosting machine | AUROC | 0.904 (0.019) | 0.807 (0.010) | - |  | 0.872 (0.009) | 0.696 (0.006) | - |
|  |  | AUPRC | 0.440 (0.042) | 0.648 (0.024) | - |  | 0.465 (0.026) | 0.528 (0.008) | - |
|  |  | F-0.5 | 0.543 (0.052) | 0.634 (0.018) | - |  | 0.521 (0.029) | 0.522 (0.006) | - |
|  |  | PPV | 0.730 (0.142) | 0.684 (0.035) | - |  | 0.598 (0.035) | 0.528 (0.017) | - |
|  |  | TPR | 0.292 (0.056) | 0.506 (0.084) | - |  | 0.347 (0.041) | 0.508 (0.044) | - |
|  | Random forest | AUROC | 0.898 (0.021) | 0.793 (0.015) | - |  | 0.861 (0.006) | 0.702 (0.004) | - |
|  |  | AUPRC | 0.345 (0.071) | 0.627 (0.024) | - |  | 0.397 (0.013) | 0.528 (0.006) | - |
|  |  | F-0.5 | 0.435 (0.061) | 0.610 (0.029) | - |  | 0.451 (0.016) | 0.518 (0.005) | - |
|  |  | PPV | 0.459 (0.095) | 0.645 (0.050) | - |  | 0.500 (0.020) | 0.529 (0.026) | - |
|  |  | TPR | 0.405 (0.093) | 0.515 (0.062) | - |  | 0.334 (0.058) | 0.491 (0.071) | - |
|  | Neural decision forest | AUROC | 0.898 (0.019) | 0.802 (0.011) | - |  | 0.854 (0.004) | 0.681 (0.003) | - |
|  |  | AUPRC | 0.464 (0.051) | 0.645 (0.024) | - |  | 0.444 (0.008) | 0.503 (0.008) | - |
|  |  | F-0.5 | 0.584 (0.051) | 0.631 (0.018) | - |  | 0.494 (0.010) | 0.507 (0.005) | - |
|  |  | PPV | 0.717 (0.135) | 0.677 (0.039) | - |  | 0.561 (0.054) | 0.509 (0.018) | - |
|  |  | TPR | 0.361 (0.064) | 0.507 (0.064) | - |  | 0.356 (0.065) | 0.507 (0.055) | - |
|  | Logistic regression | AUROC | 0.874 (0.022) | 0.790 (0.007) | - |  | 0.757 (0.032) | 0.607 (0.007) | - |
|  |  | AUPRC | 0.411 (0.038) | 0.627 (0.014) | - |  | 0.259 (0.032) | 0.433 (0.011) | - |
|  |  | F-0.5 | 0.515 (0.048) | 0.625 (0.019) | - |  | 0.296 (0.042) | 0.437 (0.015) | - |
|  |  | PPV | 0.632 (0.067) | 0.658 (0.036) | - |  | 0.284 (0.054) | 0.437 (0.028) | - |
|  |  | TPR | 0.301 (0.046) | 0.540 (0.089) | - |  | 0.422 (0.122) | 0.450 (0.065) | - |
