# Supplementary material for: Enhancing Patient Selection in Sepsis Clinical Trials Design Through an AI Enrichment Strategy: Algorithm Development and Validation
Source: J Med Internet Res. 2024 Sep 4;26:e54621. doi: 10.2196/54621 (PMC11411223; doi:10.2196/54621)
Supplement: Multimedia Appendix 7 [file jmir_v26i1e54621_app7.docx]

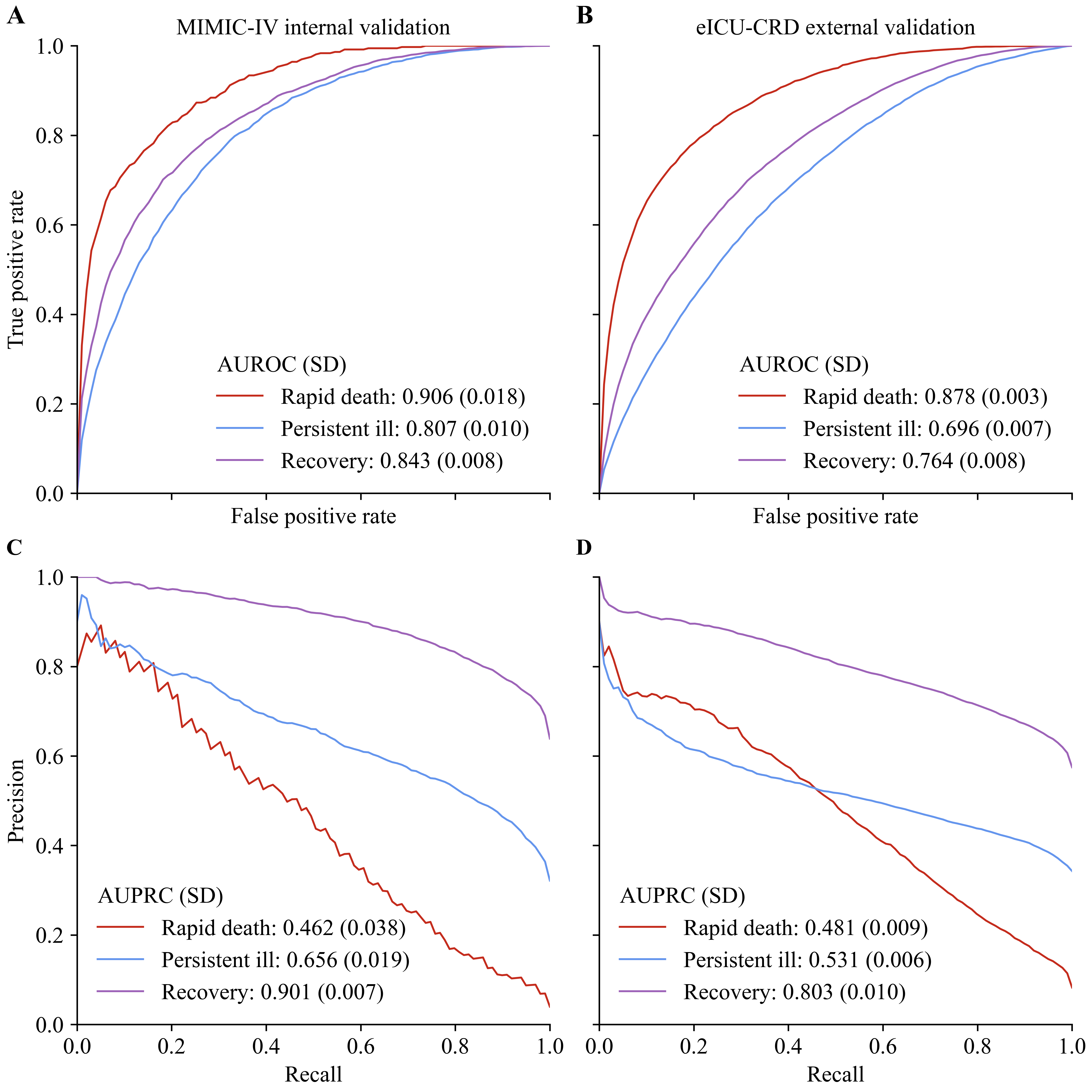


**Performance of the 3-way multiclass gradient boosting machine model using all the features for each outcome. (**A, B) Receiver operating characteristic curves. (C, D) Precision-recall curves. AUROC=the area under the receiver operating characteristic curve. AUPRC=the area under the precision-recall curve. MIMIC-IV=medical information mart for intensive care database-IV. eICU-CRD=eICU collaborative research database.
