# Supplementary material for: Enhancing Patient Selection in Sepsis Clinical Trials Design Through an AI Enrichment Strategy: Algorithm Development and Validation
Source: J Med Internet Res. 2024 Sep 4;26:e54621. doi: 10.2196/54621 (PMC11411223; doi:10.2196/54621)
Supplement: Multimedia Appendix 8 [file jmir_v26i1e54621_app8.docx]

| **Features** | **Metrics** | **MIMIC-IV internal validation** | | |  | **eICU-CRD external validation** | | |
| --- | --- | --- | --- | --- | --- | --- | --- | --- |
|  |  | **Rapid death** | **Persistent ill** | **Recovery** |  | **Rapid death** | **Persistent ill** | **Recovery** |
| All features  (148 features) | AUROC | 0.906 (0.018) | 0.807 (0.010) | 0.843 (0.008) |  | 0.878 (0.003) | 0.696 (0.007) | 0.764 (0.008) |
|  | AUPRC | 0.462 (0.038) | 0.656 (0.019) | 0.901 (0.007) |  | 0.481 (0.009) | 0.531 (0.006) | 0.803 (0.010) |
|  | F-0.5 | 0.494 (0.038) | 0.611 (0.015) | 0.813 (0.009) |  | 0.518 (0.017) | 0.511 (0.009) | 0.726 (0.007) |
|  | PPV | 0.608 (0.082) | 0.618 (0.017) | 0.805 (0.013) |  | 0.612 (0.040) | 0.522 (0.010) | 0.711 (0.012) |
|  | TPR | 0.306 (0.073) | 0.589 (0.036) | 0.848 (0.020) |  | 0.331 (0.054) | 0.478 (0.044) | 0.792 (0.027) |
|  | Brier score | 0.029 (0.001) | 0.171 (0.007) | 0.158 (0.004) |  | 0.057 (0.001) | 0.214 (0.009) | 0.203 (0.010) |
| Bortua selection  (50 features) | AUROC | 0.901 (0.019) | 0.790 (0.016) | 0.830 (0.014) |  | 0.865 (0.007) | 0.674 (0.010) | 0.751 (0.006) |
|  | AUPRC | 0.448 (0.062) | 0.630 (0.026) | 0.892 (0.010) |  | 0.443 (0.015) | 0.508 (0.014) | 0.790 (0.008) |
|  | F-0.5 | 0.464 (0.085) | 0.596 (0.019) | 0.812 (0.010) |  | 0.484 (0.023) | 0.492 (0.011) | 0.722 (0.005) |
|  | PPV | 0.541 (0.099) | 0.596 (0.020) | 0.808 (0.013) |  | 0.546 (0.043) | 0.486 (0.015) | 0.721 (0.007) |
|  | TPR | 0.303 (0.070) | 0.597 (0.036) | 0.829 (0.019) |  | 0.338 (0.030) | 0.516 (0.030) | 0.732 (0.029) |
|  | Brier score | 0.030 (0.002) | 0.178 (0.012) | 0.166 (0.011) |  | 0.060 (0.002) | 0.225 (0.011) | 0.212 (0.009) |
| Top 15 features | AUROC | 0.896 (0.017) | 0.776 (0.011) | 0.823 (0.011) |  | 0.850 (0.005) | 0.646 (0.011) | 0.744 (0.006) |
|  | AUPRC | 0.431 (0.051) | 0.605 (0.016) | 0.887 (0.009) |  | 0.391 (0.013) | 0.476 (0.010) | 0.780 (0.006) |
|  | F-0.5 | 0.455 (0.054) | 0.579 (0.014) | 0.806 (0.012) |  | 0.430 (0.020) | 0.477 (0.008) | 0.719 (0.004) |
|  | PPV | 0.504 (0.081) | 0.578 (0.013) | 0.805 (0.016) |  | 0.436 (0.038) | 0.461 (0.010) | 0.740 (0.010) |
|  | TPR | 0.358 (0.087) | 0.586 (0.039) | 0.811 (0.019) |  | 0.423 (0.057) | 0.557 (0.031) | 0.649 (0.033) |
|  | Brier score | 0.054 (0.035) | 0.189 (0.016) | 0.208 (0.059) |  | 0.084 (0.028) | 0.228 (0.010) | 0.242 (0.031) |
| NEE (max) | AUROC | 0.746 (0.039) | 0.664 (0.029) | 0.751 (0.014) |  | 0.689 (0.025) | 0.561 (0.006) | 0.667 (0.007) |
|  | AUPRC | 0.156 (0.033) | 0.454 (0.032) | 0.820 (0.015) |  | 0.159 (0.008) | 0.391 (0.007) | 0.703 (0.003) |
|  | F-0.5 | 0.163 (0.037) | 0.469 (0.016) | 0.774 (0.011) |  | 0.204 (0.021) | 0.396 (0.019) | 0.672 (0.014) |
|  | PPV | 0.143 (0.033) | 0.486 (0.029) | 0.780 (0.008) |  | 0.185 (0.019) | 0.397 (0.018) | 0.696 (0.007) |
|  | TPR | 0.366 (0.083) | 0.419 (0.042) | 0.754 (0.045) |  | 0.343 (0.062) | 0.405 (0.070) | 0.600 (0.068) |
|  | Brier score | 0.106 (0.015) | 0.212 (0.005) | 0.286 (0.030) |  | 0.126 (0.010) | 0.225 (0.002) | 0.284 (0.013) |

AUROC=area under the receiver operating characteristic curve. AUPRC=the area under the precision-recall curve. PPV=positive predictive value. TPR=true positive rate
