# Supplementary material for: Enhancing Patient Selection in Sepsis Clinical Trials Design Through an AI Enrichment Strategy: Algorithm Development and Validation
Source: J Med Internet Res. 2024 Sep 4;26:e54621. doi: 10.2196/54621 (PMC11411223; doi:10.2196/54621)
Supplement: Multimedia Appendix 9 [file jmir_v26i1e54621_app9.docx]

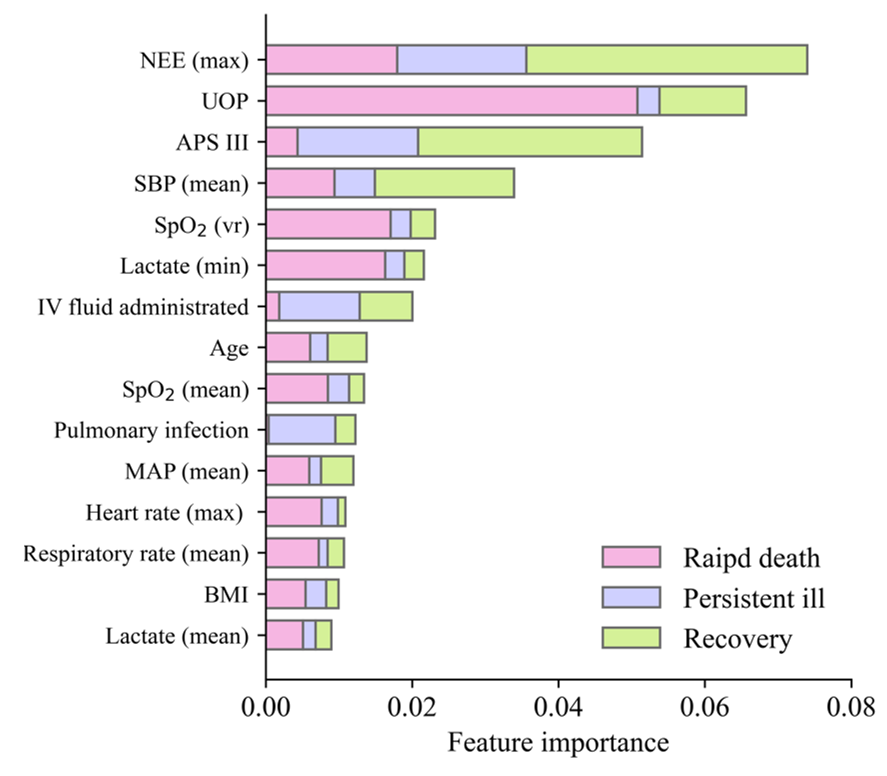


**The overall global feature importance for the model using all the features.** APS: Acute Physiology Score; BMI: body mass index; IV: intravenous; MAP: mean arterial pressure; NEE: norepinephrine equivalence; SBP: systolic blood pressure; SpO2: oxygen saturation; UOP: urine output.
